# Online Dialectical Behavioral Therapy for Emotion Dysregulation in People With Chronic Pain: A Randomized Clinical Trial

**DOI:** 10.1001/jamanetworkopen.2025.6908

**Published:** 2025-05-06

**Authors:** Nell Norman-Nott, Nancy E. Briggs, Negin Hesam-Shariati, Chelsey R. Wilks, Jessica Schroeder, Ashish D. Diwan, Jina Suh, Jill M. Newby, Toby Newton-John, Yann Quidé, James H. McAuley, Sylvia M. Gustin

**Affiliations:** 1 NeuroRecovery Research Hub, School of Psychology, University of New South Wales, Sydney, New South Wales, Australia; 2 Centre for Pain IMPACT, Neuroscience Research Australia, Sydney, New South Wales, Australia; 3 Stats Central, Mark Wainwright Analytical Centre, University of New South Wales, Sydney, New South Wales, Australia; 4 Tori Health, St Louis, Missouri; 5 School of Computer Science and Engineering, University of Washington, Seattle; 6 Spine Service, St George Hospital Campus, Kogarah, New South Wales, Australia; 7 School of Clinical Medicine, University of New South Wales, Sydney, New South Wales, Australia; 8 Spinal Unit, Discipline of Orthopaedic Surgery and Trauma, Royal Adelaide Hospital, The University of Adelaide, Adelaide, South Australia, Australia; 9 Microsoft Research, Redmond, Washington; 10 Black Dog Institute and School of Psychology, University of New South Wales, Sydney, New South Wales, Australia; 11 Graduate School of Health, University of Technology Sydney, Sydney, New South Wales, Australia; 12 School of Health Sciences, University of New South Wales, Sydney, New South Wales, Australia

## Abstract

**Importance:**

Current therapeutic approaches are inaccessible to many people with chronic pain and frequently fail to address emotion dysregulation as a key factor in psychological comorbidity and pain intensity. An effective and accessible emotion regulation–focused intervention is needed.

**Objectives:**

To compare the efficacy of online dialectical behavioral therapy for chronic pain plus treatment as usual (iDBT-Pain) with only treatment as usual on emotion dysregulation in people with chronic pain.

**Design, Setting, and Participants:**

This 2-arm randomized clinical trial was conducted from March 2023 to September 2024 in Australia. Participants were adults with chronic pain (lasting ≥3 months) and weekly pain intensity of 3 or higher out of 10 (10 indicating worst pain), without psychotic or personality disorders, and without dementia. Eligible participants were randomly assigned (1:1 ratio) to receive either iDBT-Pain for 9 weeks or treatment as usual only. Intention-to-treat data analyses were performed between August and September 2024.

**Interventions:**

The iDBT-Pain group received 8 group-based 90-minute therapist-guided online sessions as well as an app and a handbook for self-learning. Content focused on DBT skills training, including pain science education. Participants in the treatment-as-usual group continued usual care, which consisted of treatment options that can be accessed in the community.

**Main Outcomes and Measures:**

The primary outcome was emotion dysregulation at 9 weeks after randomization. The Difficulties in Emotion Regulation Scale (score range: 18-90, with higher scores indicating higher emotion dysregulation) was used in assessment.

**Results:**

Among 89 participants (mean [SD] age, 51.5 [14.2] years; 74 females [83%]), 44 (49%) were randomly assigned to the treatment-as-usual group and 45 (51%) were randomly assigned to the iDBT-Pain group. Overall, 79 participants (89%) completed the 9-week assessment. Between-group difference in emotion dysregulation over time favored iDBT-Pain over treatment as usual at 9 weeks (−4.88; 95% CI, −9.20 to −0.55; *P* = .03; Cohen *d* = −0.46 [95% CI, −0.87 to −0.08]).

**Conclusions and Relevance:**

In this randomized clinical trial, the iDBT-Pain intervention, delivered through a self-learning and therapist-guided hybrid approach, resulted in sustained improvements in emotion dysregulation in people with chronic pain.

**Trial Registration:**

Anzctr.org.au Identifier: ACTRN12622000113752

## Key Points

**Question:**

What is the effect of the online dialectical behavioral therapy for chronic pain (iDBT-Pain) intervention on emotion dysregulation in people with chronic pain?

**Findings:**

In this randomized clinical trial with 89 participants, those receiving iDBT-Pain demonstrated significant improvement in emotion dysregulation over 9 weeks after randomization compared with those who continued treatment as usual.

**Meaning:**

The findings support the superiority of iDBT-pain over usual treatment to improve emotion dysregulation in adults with chronic pain.

## Teaser

This randomized clinical trial evaluates the efficacy of a hybrid self-directed and therapist-guided therapy for emotional dysregulation in adults with chronic pain.

## Introduction

Chronic pain, defined as pain persisting beyond 3 months,^1^ affects 20% to 30% of the population.^2,3,4,5^ Beyond its sensory experience, chronic pain is an intrinsically emotional experience^1^ associated with heightened negative emotions, including anger,^6^ worry,^7^ and low mood,^8^ alongside a diminished capacity to regulate emotions.^9^ Dysregulated emotions contribute to comorbid psychological disorders^10^; symptoms of anxiety and depression, which are present in 50% to 80% of people with chronic pain^11,12,13^; and worsening pain intensity.^14,15,16^ Treating the emotional side of chronic pain with standard interventions has proven difficult,^12,13,17^ with cognitive behavioral therapy shown as ineffective in improving mood or pain intensity compared with active control.^18^ Therefore, new approaches to enhancing the ability to manage the intensity, duration, and frequency of emotions^19^ are under increasing scrutiny.^20,21,22,23,24^ A recent systematic review and meta-analysis found that emotion regulation skills–focused interventions improve emotion dysregulation, depression symptoms, pain intensity, and pain interference in people with chronic pain.^25^

One emotion regulation–focused intervention being adapted for chronic pain is dialectical behavioral therapy (DBT). Although, DBT is primarily used to treat borderline personality disorder, evidence supports its application for diverse health conditions characterized by emotion dysregulation,^26^ including chronic pain.^23,27,28,29,30,31^ While full-format DBT spans 12 months, DBT skills training, as a key mode of treatment, has been adapted to an 8- to 10-week therapy, reducing the burden of delivery.^32^ In DBT skills training, emotion regulation is improved by encouraging emotion recognition, emotion expression, and reaction evaluation. Results of small in-person trials of DBT for people with chronic pain show promise to improve emotion dysregulation, depression, anxiety, and pain intensity.^23,29,30,33^

Online DBT for chronic pain (iDBT-Pain) is a 9-week group-based intervention using a hybrid approach of self-learning in an app and a printed handbook as well as therapist-guided online sessions in DBT skills. Self-learning is related to meaningful changes in chronic pain symptoms,^34^ potentially through feelings of empowerment to self-manage treatment.^35^ Meanwhile, guided sessions provide the opportunity to clarify and discuss concepts and are aimed at mitigating attrition.^36^ As an online intervention, iDBT-Pain improves accessibility for those with limited access to health care services^37^ and promotes convenient treatment engagement for those with restricted mobility.^38^ A pilot trial of iDBT-Pain demonstrated preliminary effectiveness to reduce emotion dysregulation and pain intensity, with an exploratory analysis indicating improved depression and anxiety symptoms.^31^ Building on these findings is the current trial, which, to our knowledge, is the first randomized clinical trial (RCT) to investigate iDBT-Pain and the first to explore an online hybrid therapist-guided and self-learning emotion regulation–focused intervention for people with chronic pain.

This trial aimed to compare the effect of iDBT-Pain plus treatment as usual (hereafter, iDBT-Pain) with treatment as usual only (hereafter, treatment as usual) on emotion dysregulation at 9 weeks after randomization. We hypothesized that participants receiving iDBT-Pain compared with those receiving treatment as usual would have significantly lower scores in emotion dysregulation, the primary outcome, at the 9-week assessment. In addition, we conducted exploratory analysis of the effects of iDBT-Pain compared with treatment as usual on emotion dysregulation at 21 weeks and on related psychological and health outcomes at 9 and 21 weeks.

## Methods

### Trial Design and Participants

This 2-arm RCT was conducted between March 2023 and September 2024. The University of New South Wales Human Ethics Committee approved the trial and its protocol (Supplement 1). Participants provided written informed consent prior to participation. We followed the Consolidated Standards of Reporting Trial (CONSORT) reporting guideline.

Online advertisements invited people with chronic pain to participate in research investigating an intervention aimed at improving their abilities to cope with and regulate distressing emotions. Potential participants completed a 2-step screening process using an online survey and a telephone call with an investigator. Eligible participants were (1) adults (aged ≥18 years) with chronic pain (≥3 months), with a weekly intensity of 3 or higher out of 10 (on a Numeric Rating Scale [NRS]; score range: 0 [no pain] to 10 [worst pain imaginable]); (2) with internet access; (3) available for the iDBT-Pain sessions; (4) not diagnosed with a psychotic or personality disorder (self-reported); (5) not diagnosed with dementia (self-reported); (6) able to speak and write in English; and (7) located in Australia. Recruitment was held from March 2023 to January 2024, and the final 21-week assessment was completed in September 2024.

### Randomization and Blinding

**Figure 1.**
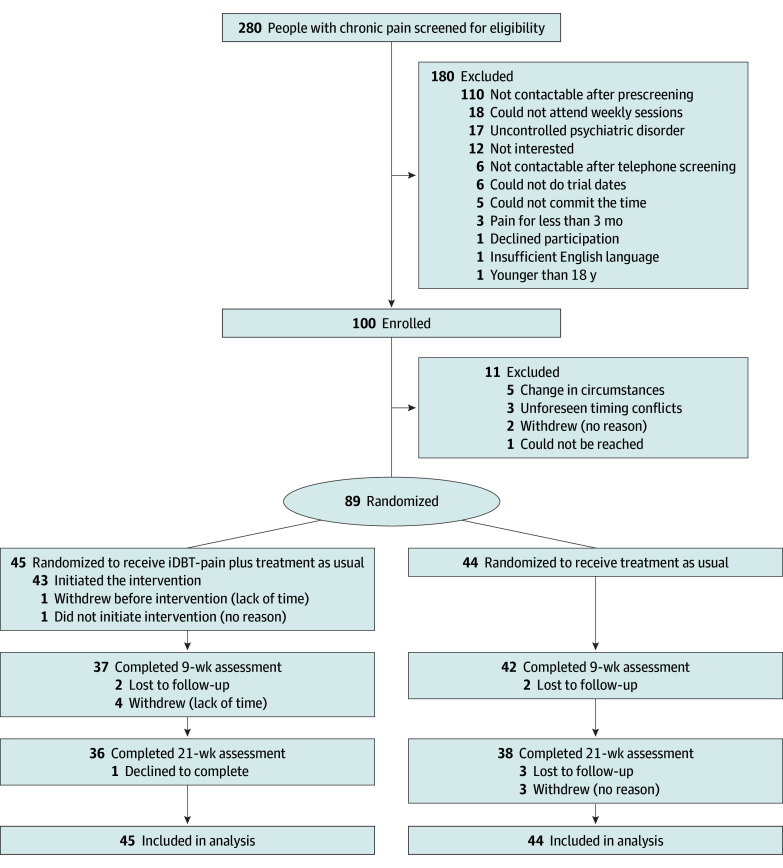
CONSORT Diagram iDBT-Pain indicates Online Dialectical Behavioral Therapy for Chronic Pain.

Participants were randomly assigned at a ratio of 1:1 to receive either iDBT-Pain (intervention group) or treatment as usual (control group) (Figure 1). A statistician, independent of the trial, generated a randomization schedule to pair participants’ unique identifier number with an assigned treatment condition. After the recruitment of each cohort of participants and completion of baseline assessment, trial clinicians and investigators learned of participants’ randomization. Participants learned of their treatment assignment by email at least 1 week prior to the first treatment session. A second statistician, responsible for analyzing the outcomes, was blinded to group assignment.

### Treatment Groups

Participants in the iDBT-Pain group attended 8 weekly 90-minute guided online sessions via videoconference, alongside self-directed learning using an app and a handbook. Content was per the protocol,^39^ in accordance with the DBT Skills Training Manual,^40^ and included pain science education (eTable 1 and eMethods in Supplement 2). Guided online sessions were led by 2 therapists, a trained psychology graduate (N.N.N.) and a qualified psychologist (S.M.G.), for groups of up to 20 participants across 3 cohorts. Session 1 was an introductory meeting, followed by 6 sessions focused on DBT skills in mindfulness (2 sessions), emotion regulation (3 sessions), and distress tolerance (1 session), with the final session consolidating all skills learned. The self-learning component used a digital app and a printed handbook, making it a blended approach that caters to individual preferences in receiving information to improve engagement.^41^ The handbook enabled deeper content absorption, note-taking, and highlighting, while the app facilitated interactive tasks and videos. Weekly text messages served as reminders to attend sessions and to practice skills daily using the app and handbook. If a participant could not attend a session live, a video recording of the missed session was provided by secure link. After the 8 sessions, participants entered the reinforcement stage whereby they continued self-learning using the app and handbook for 7 days before the 9-week assessment. After the 9-week assessment, participants had the option to continue self-learning using the app and handbook indefinitely.

Participants in the treatment-as-usual group did not receive any treatment during the trial but continued with their usual care until final assessments at 21 weeks were completed. Usual care consisted of treatment options that can be accessed in the community. At the final assessment, this group received the handbook and app.

### Data Collection

Data were collected using online questionnaires (Qualtrics), which were emailed to participants. All primary and secondary outcome measures were collected at baseline prior to randomization, at 9 weeks after randomization (primary end point), and at 21 weeks after randomization. Participants were required to complete the assessment questionnaires within 7 days of receiving them to control the assessment timeline for both the treatment-as-usual and iDBT-Pain groups. A reminder email or text message was sent to participants who did not complete the questionnaires after 5 days.

### Outcomes

The primary outcome was emotion dysregulation measured using the 18-item Difficulties in Emotion Regulation Scale (DERS-18; score range: 18-90, with higher scores indicating higher emotion dysregulation).^42^ Secondary outcomes were pain intensity, pain interference, depression symptoms, anxiety symptoms, stress, sleep problems, well-being, and posttraumatic stress symptoms (PTSS) (eMethods in Supplement 2).

### Sample Size

We based the study’s power on a pilot study—a single-case experimental design study evaluating iDBT-Pain, which demonstrated an effect size of −0.81 on pain intensity and −0.88 on emotion dysregulation.^31^ The power analysis in the trial protocol used the effect size of −0.81. However, given that single-case experimental design trials can overestimate treatment effects,^43^ we recalculated the sample size using a more conservative effect size estimate of −0.70 for the primary outcome of emotion dysregulation (see the trial protocol in Supplement 1). The sample size calculation indicated that 84 participants (42 in each group) were needed to achieve 80% power with α = .05. We allowed for a 20% attrition rate.

### Statistical Analysis

Intention-to-treat analysis was performed using a linear mixed model. The model included random effects of individuals, fixed effects of time, treatment group, and the interaction effect of time and treatment group to analyze the primary and secondary outcomes at 9 and 21 weeks. Covariates were included for age and sex. We assessed the size of the main and interaction effects by estimating the Cohen *d* scaled by the pooled baseline SD of the outcome. Effects were estimated with a 95% CI, which were bootstrapped percentile values from 1000 resamples. This model accounted for the hierarchical data structure and handled missing data under the assumption that they were missing at random.^44^ A multiple imputation sensitivity analysis was conducted to examine the robustness of the findings for the primary outcome at primary end point.

All tests were 2-sided, where *P* < .05 was considered statistically significant. Due to the potential for type I error when conducting multiple comparisons, findings for analysis of secondary outcomes and 21-week follow-up assessment should be interpreted as exploratory. All statistical analyses were completed between August and September 2024 using R, version 4.4.1 (R Project for Statistical Computing).^45^

## Results

A total of 89 participants were included (mean [SD] age, 51.5 [14.2] years; 74 females [83%], 14 males [16%], and 1 with undisclosed sex [1%]). Table 1 depicts participants characteristics and demographics at baseline. Of the 89 participants, 44 (49%) were randomly assigned to receive the control (treatment as usual) and 45 (51%) were randomly assigned to receive the intervention (iDBT-Pain) (Figure 1). Forty-three participants initiated the intervention, with 35 (81%) completing at least 7 of the 8 sessions. At the 9-week assessment, 37 participants remained in the intervention group and 42 remained in the control group. At the 21-week assessment, 36 remained in the intervention group and 38 remained in the control group. Overall, 79 participants (89%) completed the 9-week assessment, and 72 (81%) completed the 21-week assessment.

**Table 1.** Baseline Demographic and Clinical Characteristics of Participants Overall and by Treatment Group

Characteristic	Participants, No. (%)		
	Total (n = 89)	Treatment Group	
		Control (n = 44)	Intervention (n = 45)
Age, mean (SD), y	51.5 (14.2)	52.0 (15.6)	52.0 (13.8)
Sex			
Female	74 (83)	37 (84)	37 (82)
Male	14 (16)	7 (16)	7 (16)
Not disclosed	1 (1)	0	1 (1)
Ethnicity^a^^,^^b^			
Australian	66 (74)	35 (80)	31 (69)
Chinese	3 (3)	1 (2)	2 (4)
English	11 (12)	5 (11)	6 (13)
Irish	5 (6)	2 (5)	3 (7)
New Zealander	2 (2)	2 (5)	0
Scottish	5 (6)	4 (9)	1 (1)
South African	3 (3)	2 (5)	1 (1)
Other^c^	14 (16)	4 (9)	10 (22)
Occupational status			
Full-time work	15 (17)	9 (20)	6 (13)
Homemaker	5 (6)	4 (9)	1 (1)
Part-time work	23 (26)	11 (25)	12 (27)
Retired	19 (21)	13 (30)	6 (13)
Studying	5 (6)	2 (5)	3 (7)
Unemployed	2 (2)	1 (2)	1 (1)
Unknown	20 (22)	6 (14)	14 (31)
Marital status			
Divorced or separated	24 (27)	11 (25)	13 (29)
Married or living with partner	47(53)	23 (52)	24 (53)
Never been married	16 (18)	8 (18)	8 (18)
Widowed	2 (2)	2 (5)	0
Educational level			
≥Bachelor’s degree	53 (60)	24 (55)	29 (64)
High school diploma	8 (9)	5 (11)	3 (7)
Unfinished high school	4 (4)	2 (5)	2 (4)
Vocational qualification	12 (13)	6 (14)	6 (13)
Pain condition^b^			
Arthritis	33 (37)	15 (34)	18 (40)
Fibromyalgia	23 (25)	8 (18)	15 (33)
Headache	20 (22)	9 (20)	11 (24)
Lower back pain	39 (44)	21 (48)	18 (40)
Neuropathic	48 (54)	23 (52)	25 (56)
Postsurgical	13 (14)	6 (14)	7 (16)
Posttrauma	10 (11)	3 (7)	7 (16)
Other^d^	19 (21)	10 (23)	9 (20)
Pain duration, mean (SD), y	15.9 (12.8)	16.6 (13.6)	15.3 (12.2)
Taking prescribed and/or nonprescribed pain medication	68 (76)	37 (84)	31 (69)
Currently receiving psychological therapy	25 (28)	11 (25)	14 (31)

^a^ Self-reported.

^b^ Participants could report more than 1 category.

^c^ Other ethnicity includes Croatian, Sri Lankan, German, Norwegian, Turkish, Bulgarian, and Danish.

^d^ Other pain condition includes cancer pain, scoliosis, central sensitization, temporomandibular joint disorder, and visceral hyperalgesia.

### Primary Outcome

Participants in the iDBT-Pain group demonstrated a significantly greater reduction in emotion dysregulation compared with the treatment-as-usual group. The estimated interaction effect between treatment and time, assessing differential changes from baseline to 9 weeks across the groups, was −4.88 (95% CI, −9.20 to −0.55; *P* = .03), with a moderate effect indicating a meaningful and noticeable difference (Cohen *d* = −0.46; 95% CI, −0.87 to −0.08).

### Secondary Outcomes

**Figure 2.**
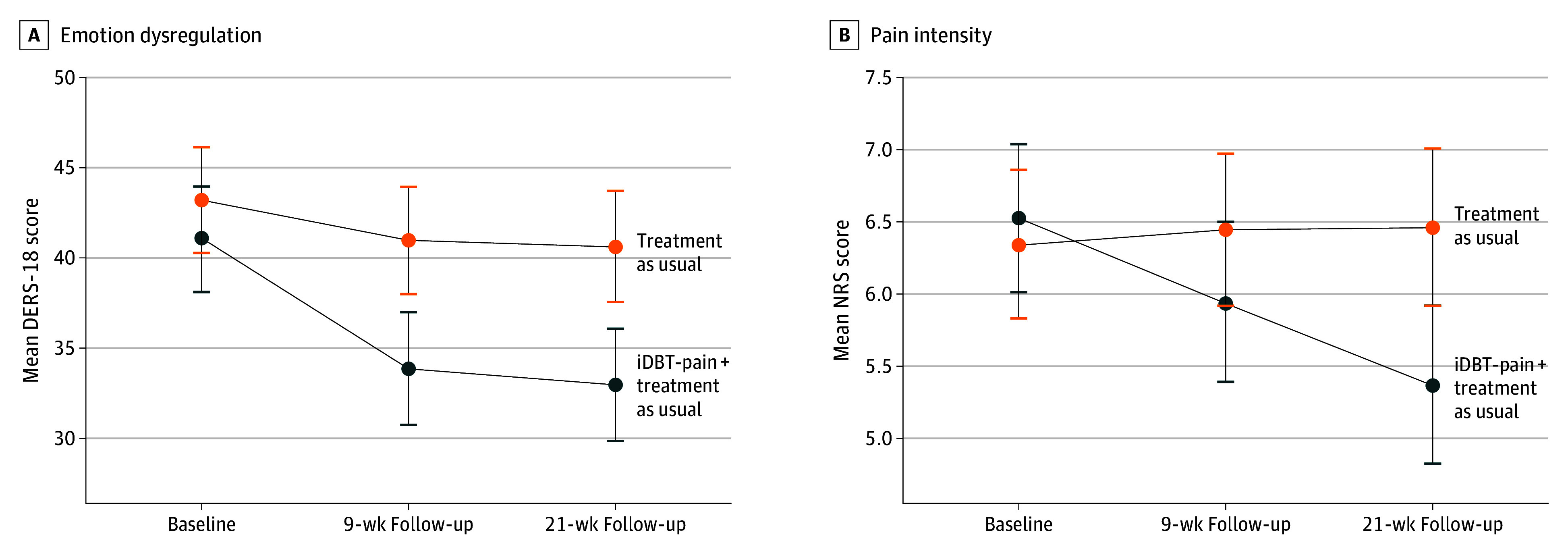
Measures of Emotion Dysregulation and Pain Intensity A, Change in the primary outcome of emotion dysregulation was measured using the 18-item Difficulties in Emotion Regulation Scale (DERS-18; score range: 18-90, with higher scores indicating higher emotion dysregulation) from baseline to 9 weeks (*P* = .03) and to 21 weeks (*P* = .02). B, Change in pain intensity was measured using the Numeric Rating Scale (NRS; score range: 0 [no pain] to 10 [worst pain imaginable]) from baseline to 9 weeks (*P* = .08) and to 21 weeks (*P* = .002). Estimated means were derived from the linear mixed models.

Table 2 shows the estimates for the interaction effect between treatment and time, assessing for differential changes between the groups from baseline to 9 weeks and from baseline to 21 weeks for all outcomes. At 21 weeks, the change in emotion dysregulation continued to favor the group receiving iDBT-Pain, with a mean difference of −5.35 points (95% CI, −9.78 to −0.97 points), indicating a moderate effect and a noticeable improvement. While no significant difference in pain intensity was observed at 9 weeks, at 21 weeks, the intervention group showed a significant improvement in pain intensity compared with treatment as usual, with a large and clinically meaningful mean difference of −1.31 points (95% CI, −2.12 to −0.49 points)^46^ (Figure 2).

**Table 2.** Summary Statistics and Linear Mixed-Model Analysis of Primary and Secondary Outcomes

Outcome, time point	Scores, Mean (SD), points				Difference in the change between groups		
	Control group	Participants, No.	Intervention group	Participants, No.	Estimate (95% CI)^a^	Cohen *d* (95% CI)^b^	*P* value
**Primary outcome**							
Emotion dysregulation (DERS-18)							
Baseline	43.2 (11.1)	44	41.0 (10.2)	45	NA	NA	NA
9-wk Assessment	40.9 (11.2)	42	34.2 (7.3)	37	−4.88 (−9.20 to −0.55)	−0.46 (−0.87 to −0.08)	.03
**Secondary outcomes**							
Emotion dysregulation (DERS-18)							
Baseline	43.2 (11.1)	44	41.0 (10.2)	45	NA	NA	NA
21-wk Assessment	40.6 (10.9)	38	33.3 (7.6)	36	−5.38 (−9.78 to −0.97)	−0.51 (−0.91 to −0.10)	.02
Depression symptoms (BDI)							
Baseline	19.3 (10.6)	44	19.0 (10.4)	45	NA	NA	NA
9-wk Assessment	23.3 (13.4)	42	14 (11.7)	37	−8.82 (−13.89 to −3.74)	−0.84 (−1.09 to −0.31)	<.001
21-wk Assessment	23.5 (18.0)	38	16.2 (12.4)	36	−7.14 (−12.33 to −1.96)	−0.68 (−0.95 to −0.17)	.007
Anxiety symptoms (SAI)							
Baseline	47.7 (14.2)	44	49.8 (11.7)	45	NA	NA	NA
9-wk Assessment	48.2 (13.3)	42	41.6 (13.7)	37	−8.41 (−13.82 to −3.00)	−0.65 (−1.19 to −0.27)	.003
21-wk Assessment	46.2 (5.4)	38	45.6 (4.17)	36	−2.81 (−8.34 to 2.72)	−0.22 (−0.69 to 0.24)	.32
Stress (NIHTB-PSS)							
Baseline	58.3 (8.7)	44	59.4 (8.7)	45	NA	NA	NA
9-wk Assessment	57.2 (9.7)	42	53.0 (7.5)	37	−5.62 (−9.27 to −1.97)	−0.65 (−1.06 to −0.24)	.003
21-wk Assessment	56.7 (8.8)	38	54.5 (7.4)	36	−3.45 (−7.18 to 0.29)	−0.40 (−0.80 to 0.03)	.07
PTSS (PCL-C)							
Baseline	44.3 (13.0)	44	44.2 (12.0)	45	NA	NA	NA
9-wk Assessment	43.8 (14.3)	42	36.4 (12.3)	37	−6.95 (−11.75 to −2.15)	−0.56 (−0.88 to −0.16)	.005
21-wk Assessment	40.2 (14.9)	38	35.9 (14.8)	36	−3.20 (−8.05 to 1.66)	−0.26 (−0.61 to 0.13)	.20
Well-being (COMPAS-W)							
Baseline	83.5 (12.6)	44	83.7 (10.7)	45	NA	NA	NA
9-wk Assessment	83.0 (13.2)	42	89.3 (10.3)	37	5.76 (0.94 to 10.59)	0.50 (0.11 to 0.86)	.02
21-wk Assessment	85.3 (14.6)	38	88.1 (11.4)	36	2.44 (−2.50 to 7.38)	0.21 (−0.22 to 0.63)	.33
Sleep problems (MOS-SS)							
Baseline	43.6 (14.7)	44	42.4 (16.9)	45	NA	NA	NA
9-wk Assessment	45.2 (12.8)	42	37.4 (14.2)	37	−6.79 (−13.18 to −0.40)	−0.43 (−0.87 to −0.04)	.04
21-wk Assessment	42.3 (14.8)	38	37.8 (11.4)	36	−3.55 (−10.08 to 2.99)	−0.23 (−0.72 to 0.22)	.29
Pain intensity (NRS)							
Baseline	6.3 (1.4)	44	6.5 (1.4)	45	NA	NA	NA
9-wk Assessment	6.4 (1.7)	42	5.9 (2.1)	37	−0.71 (−1.51 to 0.09)	−0.51 (−0.83 to 0.02)	.08
21-wk Assessment	6.4 (2.0)	38	5.3 (2.0)	36	−1.31 (−2.12 to −0.49)	−0.95 (−1.16 to −0.28)	.002
Pain interference (PROMIS)							
Baseline	64.5 (5.7)	44	64.6 (5.7)	45	NA	NA	NA
9-wk Assessment	64.8 (6.0)	42	63.3 (7.8)	37	−1.46 (−4.48 to 1.56)	−0.26 (−0.69 to 0.24)	.34
21-wk Assessment	63.9 (7.1)	38	62.7 (6.4)	36	−1.27 (−4.36 to −1.81)	−0.22 (−0.68 to 0.24)	.42

Abbreviations: BDI, Beck Depression Inventory (score range, 0-63, higher scores indicate greater depression symptoms); COMPAS-W, Composure, Own-Worth, Mastery, Positivity, Achievement, and Satisfaction Well-being Scale (score range, 26-130; higher scores indicate better well-being); DERS-18, 18-item Difficulties in Emotion Regulation Scale (score range, 18-90; higher scores indicate higher emotion dysregulation); MOS-SS, Medical Outcomes Study Sleep Scale (score range, 0-100; higher scores indicate greater sleep problems); NA, not applicable; NIHTB-PSS, National Institutes of Health Toolbox–Perceived Stress Scale (standardized score range, 22.7-87.1; higher scores indicate greater perceived stress); NRS, Numeric Rating Scale (range, 0 [no pain] to 10 [worst pain imaginable]); PCL-C, Posttraumatic Stress Disorder Checklist–Civilian (score range, 17-85; higher scores indicate greater symptoms); PROMIS, Patient-Reported Outcomes Measurement Information System Pain Interference (standardized score range, 40.9-76.8; higher scores indicate greater pain interference); PTSS, posttraumatic stress symptoms; SAI, State Anxiety Inventory (score range, 20-80; higher scores indicate greater anxiety symptoms).

^a^ Group difference in the change from baseline from the linear mixed model (group × time interaction), including all available data.

^b^ Effect size estimates calculated from scaling the pooled baseline SD of the outcome with CIs estimated from bootstrapped percentile values from 1000 resamples.

At 9 and 21 weeks, the change in depression symptoms significantly favored the iDBT-Pain group and represented an improvement from moderate depression to mild mood disturbance.^47^ The effect size estimates for depression symptoms at 9 weeks (Cohen *d* = −0.84; 95% CI, −1.09 to −0.31) and 21 weeks (Cohen *d* = −0.68; 95% CI, −0.95 to −0.17) were large and indicative of a clinically meaningful change. At 9 weeks, the differential change in anxiety symptoms significantly favored the intervention group, with a large effect size indicating a clinically meaningful improvement (Cohen *d* = −0.65; 95% CI, −1.09 to −0.31); however, the difference in anxiety symptoms was no longer statistically significant at 21 weeks. Similarly, while stress, PTSS, well-being, and sleep problems demonstrated significant differences between the groups, favoring iDBT-Pain at 9 weeks, the differences were not significant at 21 weeks. The magnitude of the difference in these outcomes at 9 weeks was moderate, indicating a noticeable and meaningful improvement (stress: Cohen *d* = −0.65 [95% CI, −1.06 to −0.24]; PTSS: Cohen *d* = −0.56 [95% CI, −0.88 to −0.16]; well-being: Cohen *d* = 0.50 [95% CI, 0.11-0.86]; sleep problems: Cohen *d* = −0.43 [95% CI, −0.87 to −0.04]). For pain interference, the differential change between the groups at both 9 weeks and 21 weeks was not significant.

Within- and between-group estimates for primary and secondary outcomes are shown in eTables 2 and 3 in Supplement 2. Multiple imputation sensitivity analysis for the primary outcome at primary end point is described in the eResults in Supplement 2.

### Adverse Events

Three of the 45 participants (7%) in the intervention group reported adverse events related to a preexisting medical condition. None of the adverse events were serious, and none were associated with study participation.

## Discussion

In this trial, iDBT-Pain resulted in a significantly greater reduction in emotion dysregulation compared with treatment as usual alone at 9 weeks, with effects sustained at 21 weeks. The benefits of iDBT-Pain extended to symptoms of depression and anxiety, alongside improvements in stress, PTSS, sleep problems, and well-being after the intervention. At the 21-week assessment, completing the intervention was associated with improvement in depression symptoms and a reduction in pain intensity. These findings are in line with those of a pilot trial of the same intervention,^31^ reinforcing the evidence that incorporating iDBT-Pain into the psychological treatment model for chronic pain is more beneficial than usual treatment. Moreover, mostly medium- or large-effect estimates across a clinically diverse sample indicate a potential for substantial clinical improvement in key psychological outcomes and pain intensity regardless of chronic pain condition.

This trial is unique in assigning emotion dysregulation as its primary outcome. Although guidelines for clinical trials of chronic pain treatment recommend measuring emotional functioning,^48^ prior RCTs of emotion regulation–focused interventions rarely measured emotion dysregulation.^25^ However, emotion dysregulation is increasingly a key target of psychological treatment in people with chronic pain,^49^ with clinical observations emphasizing its relevance in therapeutic practice for people with chronic pain.^27,50^ Emotion dysregulation is implicated in worsening psychological outcomes (eg, depression and anxiety symptoms),^11,12,13^ alongside social, occupational, and interpersonal relationship difficulties.^10,51^ Evidence points to adversity as a key factor in emotion dysregulation,^52^ indicating that the adverse experience of persistent pain serves as a continuous stressor that weakens emotion regulation abilities.^10^ Accordingly, emotion dysregulation may be a key outcome of assessment in people with chronic pain, particularly in trials evaluating psychological interventions.

To our knowledge, this trial is the first investigation of an online hybrid therapist-guided and self-learning emotion regulation–focused intervention for people with chronic pain, building on in-person approaches^20,21,22,24^ and evidence for in-person DBT.^23,27,28,29,30,31^ However, iDBT-Pain is more accessible than in-person approaches.^53^ Frequently, individuals with chronic pain experience mobility issues and a lack of health care services in rural and nonurban areas,^36,37,38^ limiting access to adequate treatment, which exacerbates both psychological and physical challenges.^37^ Using a multimodal delivery model—website, mobile, and print—iDBT-Pain seeks to overcome these common barriers by delivering online guided sessions, app-based videos, handbook articles, and video recordings of missed sessions. Meanwhile, the hybrid approach consisting of self-learning (ie, app and handbook) and therapist-guided learning (ie, online sessions) supports self-management of treatment,^34^ which has been implicated in meaningful changes in chronic pain symptoms.^35^ Additionally, guided learning stimulates discussion to maintain participation and increases content engagement.^35^ This hybrid format has been associated with strong adherence and low dropout levels^54^ compared with standard online interventions,^55^ indicating the appropriateness of the intervention for this population. However, it should be noted that a comparison of emotion dysregulation severity in the cohort vs in a general population with chronic pain was not conducted. Further investigation may be necessary to determine whether iDBT-Pain is effective for all people with chronic pain.

The results of this RCT reflect a 1-point change on a 0-to-10 NRS of pain intensity, which is a clinically meaningful between-group difference in pain^46^ and in line with in-person emotion regulation–focused interventions.^20,21,22,24^ The difference in pain intensity at the 21-week assessment, but not at 9 weeks, may indicate that emotion regulation skills need continual practice to elicit a meaningful reduction. However, because pain intensity at 21 weeks was a secondary outcome, and given that the differences in the change between groups over time in almost all other outcomes were not reduced at follow-up, this finding should be treated with caution. An ongoing change in depression symptoms aligns with the findings of a meta-analytic review,^25^ supporting the notion that emotion regulation–focused interventions alleviate co-occurring depressive symptoms. Emotion dysregulation is implicated in the development and maintenance of depression,^56^ symptoms of which are present in up to 80% of people with chronic pain.^12^ Improving emotion dysregulation may therefore be a necessary task in the therapeutic model for chronic pain to provide sustained changes in this pervasive outcome. Improvements in anxiety symptoms, stress, PTSS, sleep problems, and well-being indicate tangible changes in key symptoms and lifestyle factors that are present in people with chronic pain. However, it should be noted that improvement in these factors was short term.

### Limitations

This study has some limitations. First, the predominantly female sample highlights the need to recruit more representative groups in subsequent trials.^57^ Second, the high socioeconomic status of participants may have enhanced digital literacy, potentially affecting outcomes and underscoring the need for more recruitment across diverse socioeconomic backgrounds. Third, a 12-month follow-up would provide insights into long-lasting effects and should be considered for future trials. Fourth, as neither the therapists nor participants were blinded to the treatment condition, we cannot exclude treatment expectancy effects. Fifth, while this study compared iDBT-Pain with treatment as usual, it is unknown whether iDBT-Pain outperforms other established interventions. Future research should compare iDBT-Pain with active evidenced-based treatments, such as cognitive behavioral therapy, to determine the relative effectiveness of iDBT-Pain. Sixth, while pretrial consumer engagement identified emotion dysregulation as a major concern and was the rationale for its selection as the primary outcome of this trial, the Initiative on Methods, Measurement, and Pain Assessment in Clinical Trials recommends pain intensity as the primary outcome for chronic pain trials.^48^ Since pain intensity was a secondary outcome in the current trial, the related findings are preliminary, and future trials of iDBT-Pain should prioritize pain intensity as a primary outcome. Seventh, recruitment materials may have attracted participants with heightened emotional difficulties, which could limit the generalizability of findings to the broader population with chronic pain. Eighth, the high number of secondary outcomes in this trial increases the risk of type I error, highlighting the need for cautious interpretation of the findings. Ninth, given the interventions grounding in DBT, which primarily targets emotion dysregulation, it may require modifications to directly address pain-specific concerns.

## Conclusions

In this RCT of an emotion regulation–focused intervention (iDBT-Pain) delivered through a hybrid of therapist-guided and self-learning methods, significant and sustained improvement in emotion dysregulation was found in people with chronic pain. Additionally, iDBT-Pain provided sustained improvement in depression symptoms and a clinically significant reduction in pain intensity. Short-term improvements in anxiety symptoms, stress, PTSS, sleep problems, and well-being indicated that the benefits of iDBT-Pain extended to the adverse effects of chronic pain on key mental health and lifestyle factors.

## Notes

Supplement 1.
Trial Protocol

Supplement 2.
eMethods.
**eTable 1.** Online Dialectical Behavioral Therapy Session Details and Homework Tasks
eResults.
**eTable 2.** Between Group Difference at 9- and 21-Weeks **eTable 3.** Within Group Change at 9- and 21-Weeks
eReferences

Supplement 3.
Data Sharing Statement
