# Supplementary material for: Online Dialectical Behavioral Therapy for Emotion Dysregulation in People With Chronic Pain: A Randomized Clinical Trial
Source: JAMA Netw Open. 2025 May 6;8(5):e256908. doi: 10.1001/jamanetworkopen.2025.6908 (PMC12056567; doi:10.1001/jamanetworkopen.2025.6908)
Supplement: Supplement 1. — Trial Protocol [file jamanetwopen-e256908-s001.pdf]

Clinical Trial Protocol

Physiological, Psychological, Psychiatric, Surgical or Health Interventions

Internet Delivered Skills Training for Chronic Pain (iDBT-Pain) – A Pilot Investigation

Version 6, August 2023

A/Prof Sylvia Gustin

Contents

1. General Information ... 3
2. Safety and Monitoring Contacts ... 5
3. Trial Objectives and Purpose ... 7
4. Background Information ... 7
5. Statement of Compliance ... 11
6. Trial Design ... 11
7. Sample Size ... 13
8. Selection and Withdrawal of Subjects ... 13
   8.1 Inclusion Criteria ... 13
   8.2 Exclusion Criteria ... 13
   8.3 Recruitment Strategy ... 14
   8.4 Screening ... 14
   8.5 Consent ... 16
   8.6 Withdrawal of Consent or Participant ... 16
9. Treatment of Subjects ... 17
10. Safety and Monitoring ... 25
11. Non-compliance, Protocol Deviation and Serious Breaches of Good Clinical Practice ... 31
   11.1 Protocol Deviation ... 31
   11.2 Serious Breach of Good Clinical Practice ... 32
   11.3 Reporting Protocol Deviations ... 32
   11.4 Reporting of a Serious Breach ... 32
   11.5 Reporting of Serious Breaches by Third Parties ... 33
12. Review of a Protocol Deviation and a Serious Breach ... 33
13. Statistics ... 33
14. Data Ownership ... 34
15. Handling and Reporting Data ... 34
16. Monitoring Quality Control and Quality Assurance ... 35
17. Clinical Trial Research Agreement ... 36
18. Research Governance Site Authorisation ... 36
19. Good Clinical Practice Requirements ... 36
20. Essential Documents for the Conduct of a Clinical Trial ... 36
21. Clinical Trial Delegation and Responsibilities Log ... 37
22. Safety Monitoring Register ... 41

General Information

**Protocol Title**			
**Protocol identifying number**	**HC220078**		
**Version Number**	**6**	**Version date**	**23/08/2023**
**Amendment History**<br><br>**July 2022 -** change the data capture tool from RedCap to Qualtrics due to a number of the measures that we plan to use in this trial already being stored and being used by other researchers at UNSW within Qualtrics. Adding three further questions to the semi-structured interview to help inform further developments of the intervention. Modification submitted to ethics 29 June 2022 and approved by the HREC Executive on 7 July 2022. This change was included in the published protocol.<br><br>**Feb 2023 -** change the wording in the exclusion criteria to help clarify what conditions are excluded. Modification submitted to ethics 7 Feb 2023 and approved by the HREC Executive on 9 Feb 2023. This change was included in the published protocol.<br><br>**April 2023 -** change the independent medical expert to Dr Ashish Diwan due to incumbent no longer available due to external commitments. Modification submitted to ethics 24 April 2023 and approved by the HREC Executive on 27 April 2023.<br><br>**August 2023** – change the sample size for this trial to improve the power to detect a noticeable effect in the participants. Previously we based our power analysis on a very small study (n=3), however SCED trials can overestimate treatment effects, so we recalculated the sample size to a more conservative effect size estimate of -.07 for the primary outcome of emotion dysregulation. This change was updated on the clinical trial registration. Modification submitted to ethics 8 Aug 2023 and approved by the HREC Executive on 17 Aug 2023.			
**Version Number**		**Version date**	
**Clinical Trial Sponsor**			
**Sponsor Name**	**UNSW**		
**Sponsor Contact**	**Dr Ted Rohr**		
**Telephone**	**0417844054**		
**Email**	**ted.rohr@unsw.edu.au**		
**Address**	**UNSW, Kensington NSW 2052**		
**Coordinating Principal Investigator**			
**Name**	**Dr Sylvia Gustin – Principal Investigator**		
**Telephone**			
**Email**	**s.gustin@unsw.edu.au**		
**Type of Appointment with UNSW**	☒ UNSW Employee<br>☐ UNSW Conjoint		

	☐ Other (Please describe)			
**Principal Investigator - 1**				
**Name**	Dr Sylvia Gustin			
**Contact**	Email	s.gustin@unsw.edu.au	Telephone	
**Site**	UNSW			
**Investigator - 2**				
**Name**	Nell Norman-Nott			
**Contact**	Email	n.normannott@unsw.edu.au	Telephone	
**Site**	UNSW			
**Investigator - 3**				
**Name**	Dr Negin Hesam-Shariati			
**Contact**	Email	n.hesam_shariati@unsw.edu.au	Telephone	
**Site**	UNSW			
**Investigator - 4**				
**Name**	Dr Yann Quidé			
**Contact**	Email	y.quide@unsw.edu.au	Telephone	
**Site**	UNSW			
**Investigator - 5**				
**Name**	Pauline Zahara			
**Contact**	Email	p.zahara@unsw.edu.au	Telephone	
**Site**	**UNSW**			
**Investigator - 6**				
**Name**	Dr James McAuley			
**Contact**	Email	james.mcauley@unsw.edu.au	Telephone	
**Site**	UNSW			
**Investigator - 7**				
**Name**	Thiago Folly			
**Contact**	Email	t.folly@unsw.edu.au	Telephone	
**Site**	UNSW			
**Personnel authorised to sign the protocol and the protocol amendment(s) for the Sponsor** (ICH GCP 6.1.3)				
**Name**	Dr Sylvia Gustin – Principal Investigator			
**Telephone**				
**Email**	s.gustin@unsw.edu.au			
**Address**	School of Psychology UNSW			

**Human Research Ethics Committee**	
**Name**	**UNSW**
**Status of ethical review**	☒**Approved**<br>☐ **In progress**<br>☐**To be submitted**
**Trial Sites**	**UNSW Sydney, Level 1, BioLink Building**<br>**Neuroscience Research Australia, Randwick**
**Funding for the Clinical Trial**	
**Funding Body Name**	**N/A**

**Amount of Funding**	**N/A**
**Interests that the funding body has in the clinical trial**	**N/A**
**Insurance for Clinical Trial**	
**Insurer**	**UNSW**
**Type of Insurance**	Clinical trials are not automatically covered by UNSW insurance, and confirmation must be obtained by completing the Clinical Trials Spreadsheet and sending it to the UNSW Insurance manager (peter.mccarthy@unsw.edu.au).<br>Once insurance has been confirmed, attach a copy of the insurance certificate to the trial protocol.
**Confirmation of Insurance**	☒ **Attached**<br>☐ **In progress**<br>☐**To be submitted**

1. Safety and Monitoring Contacts

**Clinical Trials Involving Physiological, Psychological, Psychiatric or Surgical Interventions**	
**Qualified Physician/Medical Expert**	
**Name**	Dr Sylvia Gustin – APHRA Registered Psychologist –
**Telephone**	
**Email**	s.gustin@unsw.edu.au
**Address**	School of Psychology UNSW
**Sponsors Independent Physician/Medical Expert**	
**Name**	Dr Ashish Diwan – Surgeon at St Georges Hospital, Clinical Academic Senior Lecturer UNSW
**Telephone**	
**Email**	a.diwan@unsw.edu.au
**Address**	School of Clinical Medicine, UNSW
**Pharmacy, Clinical Laboratory, Radiology, Pathology and other medical and technical departments involved in the trial**	
**Name**	NeuRA Imaging
**Telephone**	02 9399 1000
**Email**	neuraimaging@neura.edu.au
**Address**	Neuroscience Research Australia, Randwick
**Independent Safety Monitoring Board or Data Safety Monitoring Board Members**	

**Trial Management Group**	
• **Associate Professor Sylvia Gustin**<br>• **Nell Norman-Nott**<br>• **Yann Quidé**<br>• **Pauline Zahara**<br>• **Thiago Folly**	
**Sponsors Independent Physician/Medical Expert**	
**Name**	Dr Ashish Diwan – Surgeon at St Georges Hospital, Clinical Academic Senior Lecturer UNSW
**Telephone**	
**Email**	a.diwan@unsw.edu.au
**Address**	School of Clinical Medicine, UNSW
**Pharmacy, Clinical Laboratory, Radiology, Pathology and other medical and/or technical departments involved in the trial**	
**Name**	NeuRA Imaging
**Telephone**	02 9399 1000
**Email**	neuraimaging@neura.edu.au
**Address**	Neuroscience Research Australia, Randwick

Delegation of Clinical Trial Duties

Responsibilities for the conduct and oversight for the trial are delegated to you as the Coordinating Principal Investigator. You may delegate trial related responsibilities to the listed Principal Investigator(s) and any trial-related personnel. All trial-related duties delegated by the Coordinating Principal Investigator or Principal Investigator(s) and trial-related personnel must only be delegated to those that are qualified by experience and training. Delegated responsibilities must be retained in the UNSW Clinical Trial Delegation Log. The UNSW Sponsor's Delegate is to be notified of the following:

- Protocol deviation reports outlined in the UNSW Research Misconduct Procedure.
- Any serious breach of Good Clinical Practice, the clinical trial protocol, the clinical trial standard operating procedures, or the human ethics approval that is likely to affect to a significant degree the safety or rights of participants or the reliability and robustness of the data generated in the clinical trial.
- Significant safety issues that are likely to (or have the potential to) affect to a significant degree the safety or rights of participants or the reliability and robustness of the data generated in the clinical trial.
- Urgent safety measures implemented to remove or prevent a significant safety issue.
- Safety reports relating to the continuation, suspension, or discontinuation of the clinical trial for safety reasons.
- Non-compliance with the protocol, SOPs, GCP, and applicable regulatory requirement(s) significantly affects or can potentially affect human subject protection or reliability of trial results significantly.
- Participant complaints or concerns received concerning the conduct of the research.

- Significant modifications to the clinical trial are likely to affect a significant degree the safety or rights of participants or the reliability and robustness of the data generated in the clinical trial.
- Addition of participating trial sites, contractual arrangements at participating sites or modifications to legal agreements.
- The intention to conduct the trial in other countries.

2. Trial Objectives and Purpose

- Describe the aim(s) of the clinical trial and specify the research questions that the trial will address.

In a recent pilot study (ethics approval: HC2001990) using a single-case experimental design (*N*=3) evidence was promising for the feasibility and acceptability of an online skills training for people in chronic pain (internet-delivered dialectical behavioural therapy skills training; iDBT-Pain) (Norman-Nott et al., 2021).

The purpose of the current pilot trial is to further investigate the effectiveness of iDBT-Pain for individuals with chronic pain to determine the effects on the emotional, sensory, and neurological components associated with chronic pain. The objectives of this trial are to:

1. Reduce emotion dysregulation
2. Reduce pain intensity
3. Improve psychological factors associated with chronic pain (e.g., symptoms of anxiety and depression, harm avoidance traits, sleep quality, life satisfaction, and coping behaviours)
4. Conduct exploratory investigation of changes in brain function (e.g., neurochemical levels in the medial prefrontal cortex)

We additionally aim to attain qualitative data to optimise the design of the intervention to provide crucial information for funding to facilitate future development and testing in a full-scale randomised controlled trial.

- Specify the primary endpoints and the secondary endpoints to be measured during the trial.

Participants will be randomly assigned to either a 'treatment' or 'treatment-as-usual' condition. It is hypothesised that participants in the 'treatment' condition will demonstrate a significant improvement in the primary outcome (emotion dysregulation) and secondary outcomes (pain intensity, symptoms of anxiety and depression, harm avoidance, sleep quality, life satisfaction, coping behaviours and social cognitive skills) when measured at post-assessment (primary endpoint) following the 9-week intervention, and this will remain when measured at 3-month follow-up (secondary endpoint).

A further secondary outcome is change in cortical function (e.g., changes in neurotransmitters' levels) which will be observed using magnetic resonance imaging at baseline and post assessment. A semi-structured interview will be used to gather information about participants experience of the skills training during the post-assessment.

3. Background Information

- Describe the theoretical background for the clinical trial and describe the disease or medical condition that the trial aims to prevent, detect, treat, or manage and provide supporting background literature references.

Emotion dysregulation frequently co-occurs with chronic pain (defined as pain lasting longer than three months (IASP, 2011), which in turn leads to heightened emotional and physical suffering (Lumley et al., 2011). Despite the availability of analgesics, pain-relieving medications have little effect on the emotional problems associated with chronic pain (Bair et al., 2003; Linton & Bergbom, 2011), and come with substantial risks for side effects and addiction (Finnerup et al., 2015). As a psychological treatment, cognitive-behavioural therapy (CBT) has no side effects, but also shows limited beneficial effect for pain intensity and emotions (Williams et al., 2020). CBT may be limited to treat chronic pain because it does not substantially address emotion regulation difficulties commonly associated with chronic pain (Koechlin et al., 2018; Lumley & Schubiner, 2019; Naylor et al., 2017). Thus, evidence is needed for psychological interventions that can be applied specifically for the emotional aspects of chronic pain, particularly emotion dysregulation (Lumley et al., 2011). It has further been shown by our group that neurological changes in the brain, specifically dysregulated neurochemicals in the medial pre-frontal cortex that are related to chronic pain, may be responsible for the co-occurrence of emotion dysregulation and subsequent limited efficacy of CBT (Naylor et al., 2019). Therefore, it is necessary to investigate the potential that emotion-focused interventions for chronic pain may improve neurological function, particularly in the medial pre-frontal cortex.

- Provide theoretical background information for the intervention. Justify the use of these interventions, how it will be administered or treatment periods by including a summary of findings from non-clinical studies. Include supporting provide background literature references.

Increasingly, dialectical-behavioural therapy (DBT), designed to improve emotion regulation capabilities, is effective for the emotional and sensory components of chronic pain (Boersma et al., 2019; Linton & Fruzzetti, 2014; Linton, 2010; Sysko et al., 2016). DBT is primarily delivered in-person, hence even-though it has been found to be effective for people with chronic pain, it's use is limited in terms of access for rural, regional, and remote areas and indigenous communities (Hogg et al., 2021). Moreover, the Covid-19 pandemic has further limited access for people in chronic pain due to closure of treatment centres and risk of infection (Eccleston et al., 2020). Internet-delivered treatment is a viable option to increase capacity to deliver psychological interventions effectively for chronic pain regardless of geographical and other restrictions (Eccleston et al., 2014). Additionally, evidence finds that DBT skills training is effective when delivered in online formats to other clinical populations (Schroeder et al., 2018). However, to our knowledge, our intervention, internet-delivered dialectical behavioral therapy skills training (iDBT-Pain) (Norman-Nott et al., 2021), is the only existing online DBT skills training for chronic pain.

The iDBT-Pain skills training utilises evidence-based protocols for DBT skills training and will be administered according to the DBT Skills Training manual (Linehan, 2015). The current trial will involve participants in the treatment condition completing an eight-week internet-delivered skills training for individuals with chronic pain (iDBT-Pain). iDBT-Pain encompasses one 60-90 minute

introductory session, six 60-90 minute iDBT-Pain sessions delivered by a DBT skills trainer via Zoom in groups of 12 participants, one concluding session, and the iDBT-Pain app accessed via a smart device which includes videos and tasks to encourage skills practice between the iDBT-Pain sessions.

Findings from a prior pilot trial conducted by our group, using a single case design with multiple baselines, demonstrated promising results for the acceptability, feasibility, and efficacy of iDBT-Pain skills training for individuals with chronic pain (Norman-Nott et al., 2021). However, to be able to apply these findings more broadly, evidence obtained from a larger group such as will be achieved with this pilot randomised control trial is necessary.

- Describe the population studied in the clinical trial and provide background literature references to justify their inclusion in the trial.

Chronic pain is a substantial and costly source of human suffering affecting one in five people (Blyth et al., 2001). Economic costs in Australia are over $139 billion annually (Puezzullo, 2019), and alarmingly 20% of people have considered suicide to end their suffering (Tang & Crane, 2006). Increasingly, difficulties in emotion regulation are related to heightened emotional and physical suffering associated with chronic pain leading to recommendations that the emotional aspects of chronic pain are targeted by psychological interventions which incorporate emotional skills training (Koechlin et al., 2018; Lumley & Schubiner, 2019; Naylor et al., 2017). Moreover, access to in-person psychological intervention are hindered in this cohort because of the restricted mobility and disability that are a common feature of chronic pain (Dansie & Turk, 2013). These factors have led to the recommendation that psychological interventions for chronic pain be delivered online (Eccleston et al., 2014). In summary, the chronic pain population may substantially benefit from this trial given the need for online emotion focused interventions for the treatment and management of the emotional and sensory components of chronic pain. Furthermore, efficacy of the iDBT-Pain skills training, is promising for the chronic pain population given the prior evidence (Norman-Nott et al., 2021).

References

1. Norman-Nott, N., Wilks, C., Hesam-Shariati, N., Schroeder, J., Suh, J., Czerwinski, M., . . . Gustin, S. M. (2021). The No-Worries trial: Evaluating the efficacy of the iDBT-Pain skills training intervention to reduce emotional dysregulation and pain intensity in people with chronic pain (under review). *The Journal of Pain*.
2. IASP. (2011). Part III Pain Terms. In H. Merskey & N. Bogduk (Eds.), *Classification of Chronic Pain*. IASP. https://s3.amazonaws.com/rdcms-iasp/files/production/public/Content/ContentFolders/Publications2/ClassificationofChronicPain/Part_III-PainTerms.pdf
3. Lumley, M. A., Cohen, J. L., Borszcz, G. S., Cano, A., Radcliffe, A. M., Porter, L. S., . . . Keefe, F. J. (2011). Pain and emotion: a biopsychosocial review of recent research. *Journal of Clinical Psychology, 67*(9), 942-968. https://doi.org/10.1002/jclp.20816
4. Bair, M. J., Robinson, R. L., Katon, W., & Kroenke, K. (2003). Depression and Pain Comorbidity: A Literature Review. *Archives of Internal Medicine, 163*(20), 2433-2445. https://doi.org/10.1001/archinte.163.20.2433
5. Linton, S. J., & Bergbom, S. (2011). Understanding the link between depression and pain. *Scand J Pain, 2*(2), 47-54. https://doi.org/10.1016/j.sjpain.2011.01.005

6. Finnerup, N. B., Attal, N., Haroutounian, S., McNicol, E., Baron, R., Dworkin, R. H., . . . Wallace, M. (2015). Pharmacotherapy for neuropathic pain in adults: a systematic review and meta-analysis. *The Lancet. Neurology, 14*(2), 162-173. https://doi.org/10.1016/S1474-4422(14)70251-0
7. Williams, A. C. d. C., Fisher, E., Hearn, L., & Eccleston, C. (2020). Psychological therapies for the management of chronic pain (excluding headache) in adults. *Cochrane Database of Systematic Reviews*(8). https://doi.org/10.1002/14651858.CD007407.pub4
8. Lumley, M. A., & Schubiner, H. (2019). Psychological Therapy for Centralized Pain: An Integrative Assessment and Treatment Model. *Psychosomatic Medicine, 81*(2), 114-124. https://doi.org/10.1097/PSY.0000000000000654
9. Koechlin, H., Coakley, R., Schechter, N., Werner, C., & Kossowsky, J. (2018). The role of emotion regulation in chronic pain: A systematic literature review. *J Psychosom Res, 107*, 38-45. https://doi.org/10.1016/j.jpsychores.2018.02.002
10. Naylor, B., Boag, S., & Gustin, S. M. (2017). New evidence for a pain personality? A critical review of the last 120 years of pain and personality. *Scandinavian Journal of Pain, 17*, 58-67.
11. Naylor, B., Hesam-Shariati, N., McAuley, J., Boag, S., Newton-John, T., Rae, C., & Gustin, S. M. (2019). Reduced glutamate in the medial prefrontal cortex is associated with emotional and cognitive dysregulation in people with chronic pain. *Frontiers in Neurology, 10*, 1110.
12. Sysko, H., Thorkelson, G., & Szigethy, E. (2016). P-001 Dialectical Behavior Therapy for Chronic Pain in Gastrointestinal Disorders: A Pilot Study. *Inflammatory Bowel Diseases, 22*(suppl_1), S9-S9. https://doi.org/10.1097/01.Mib.0000480106.40651.21
13. Linton, S. J. (2010). Applying dialectical behavior therapy to chronic pain: A case study. *Scand J Pain, 1*(1), 50-54. https://doi.org/10.1016/j.sjpain.2009.09.005
14. Linton, S., & Fruzzetti, A. (2014). A hybrid emotion-focused exposure treatment for chronic pain: A feasibility study. *Scandinavian Journal of Pain, 5*, 151-158. https://doi.org/10.1016/j.sjpain.2014.05.005
15. Boersma, K., Södermark, M., Hesser, H., Flink, I. K., Gerdle, B., & Linton, S. J. (2019). Efficacy of a transdiagnostic emotion-focused exposure treatment for chronic pain patients with comorbid anxiety and depression: a randomized controlled trial. *Pain, 160*(8), 1708-1718. https://doi.org/10.1097/j.pain.0000000000001575
16. Hogg, M. N., Kavanagh, A., Farrell, M. J., & Burke, A. L. J. (2021). Waiting in Pain II: An Updated Review of the Provision of Persistent Pain Services in Australia. *Pain medicine (Malden, Mass.), 22*(6), 1367-1375. https://doi.org/10.1093/pm/pnaa374
17. Eccleston, C., Blyth, F. M., Dear, B. F., Fisher, E. A., Keefe, F. J., Lynch, M. E., . . . Williams, A. C. C. (2020). Managing patients with chronic pain during the COVID-19 outbreak: considerations for the rapid introduction of remotely supported (eHealth) pain management services. *Pain, 161*(5), 889-893. https://doi.org/10.1097/j.pain.0000000000001885
18. Eccleston, C., Fisher, E., Brown, R., Craig, L., Duggan, G. B., Rosser, B. A., & Keogh, E. (2014). Psychological therapies (Internet-delivered) for the management of chronic pain in adults. *Cochrane Database of Systematic Reviews*(2). https://doi.org/10.1002/14651858.CD010152.pub2
19. Linehan, M. M. (2015). *DBT Skills Training Manual* (2 ed.). The Guildford Press.
20. Blyth, F. M., March, L. M., Brnabic, A. J., Jorm, L. R., Williamson, M., & Cousins, M. J. (2001). Chronic pain in Australia: a prevalence study. *Pain, 89*(2-3), 127-134. https://doi.org/10.1016/s0304-3959(00)00355-9
21. Puezzullo, L. (2019). *The Cost of Pain in Australia: Report for Pain Australia*. D. A. Economics. https://www.painaustralia.org.au/static/uploads/files/the-cost-of-pain-in-australia-launch-20190404-wfrsaslpzsnh.pdf
22. Tang, N. K., & Crane, C. (2006). Suicidality in chronic pain: a review of the prevalence, risk factors and psychological links. *Psychol Med, 36*(5), 575-586. https://doi.org/10.1017/s0033291705006859
23. Dansie, E. J., & Turk, D. C. (2013). Assessment of patients with chronic pain. *British Journal of Anaesthesia, 111*(1), 19-25. https://doi.org/10.1093/bja/aet124

24. Moore, C. G., Carter, R. E., Nietert, P. J., & Stewart, P. W. (2011). Recommendations for planning pilot studies in clinical and translational research. *Clin Transl Sci, 4*(5), 332-337. https://doi.org/10.1111/j.1752-8062.2011.00347.x
25. Neacsiu, A. D., Bohus, M., & Linehan, M. M. (2014). Dialectical behavior therapy: An intervention for emotion dysregulation. In *Handbook of emotion regulation, 2nd ed.* (pp. 491-507). The Guilford Press.
26. Gustin, S. M., Wilcox, S. L., Peck, C. C., Murray, G. M., & Henderson, L. A. (2011). Similarity of suffering: Equivalence of psychological and psychosocial factors in neuropathic and non-neuropathic orofacial pain patients. *Pain, 152*(4), 825-832. https://doi.org/10.1016/j.pain.2010.12.033

4. Statement of Compliance

The clinical trial will be conducted in compliance with the following guidelines and documentation:

- ICH Guidelines for Good Clinical Practice (GCP)
- National Statement on Ethical Conduct in Human Research (National Statement)
- As approved by the Human Research Ethics Committee (HREC), the clinical trial protocol is responsible for monitoring the trial's conduct.
- The responsibilities set out by the UNSW Sponsors Delegate.
  The onsite or remote monitoring standard operating procedures as put in place by the clinical trial sponsor.

The coordinating principal investigator accepts responsibility for the accuracy of the information provided in this application and ensures that the qualifications and/or experience of all members of the iDBT-Pain research team involved with the project are appropriate to their role. The coordinating PI will ensure that all tasks will only be delegated to appropriately trained, skilled, and qualified staff. The PI will remain responsible for the overall study conduct and reported data, ensuring study oversight. All associates, colleagues, and employees assisting in the conduct of the study will be informed about their obligations and will not perform any study tasks before appropriate delegation and completion of appropriate training. All research staff will receive the appropriate information and training throughout the study and a 2-way communication channel will exist between staff and the PI. Any delegation of staff or changes in staff will be recorded promptly in the Clinical Trial Delegation and Responsibilities Log (see 22. Clinical Trial Delegation and Responsibilities Log). iDBT-Pain researchers will maintain valid ICH Good Clinical Practice certification for the duration of the trial. Sylvia Gustin, Nell Norman-Nott, Negin Hesam-Shariati, Yann Quide, and Pauline Zahara have completed the “Introduction to Good Clinical Practice” Course. (Certificates are filed in the eTMF [electronic Trial Master File]).

5. Trial Design

- Describe the selected trial design (e.g., double-blind, placebo-controlled, parallel design) and justify how it will meet the clinical trial aims.

This trial is a pilot randomised controlled trial with participants with chronic pain randomised to one of two arms: ‘treatment’ and ‘treatment-as-usual’. Efficacy of iDBT-Pain will be determined by comparing treatment to treatment-as-usual for the primary and secondary outcomes. Treatment-as-usual is defined as any of the treatment options offered by the healthcare professionals’ that participants would normally access in the community.

- Describe the measures to be implemented to minimise and avoid bias (e.g. randomisation, blinding)

Forty-eight participants will be recruited to the study (see 6. Sample Size). Following informed consent, and before baseline assessment participants will be randomly allocated to groups (treatment or treatment-as-usual) in blocks of 6. A randomisation schedule will be created in Excel by the statistician who is not involved in the recruitment, treatment, or data collection to minimise risk of bias.

Allocation of participants to randomisation schedule: Participants will have been assigned a participant ID in Qualtrics during the study screening process (see Section 7.4). This ID will be provided to a member of the research team to be added in the next available cell in the randomisation schedule Excel sheet. The member of the research team that adds the participant ID to randomisation schedule Excel sheet will not have access to any information about the participant except the participant ID and will not be involved in the recruitment, treatment, or data collection. Once the participant has been added to the Excel sheet, the team member will advise the skills trainer of the participants group allocation and the group allocation will be updated by the skills trainer in Qualtrics. The skills trainer will communicate the group allocation to each participant by phone or email.

- Provide a schematic diagram of trial design, procedures, and stages.

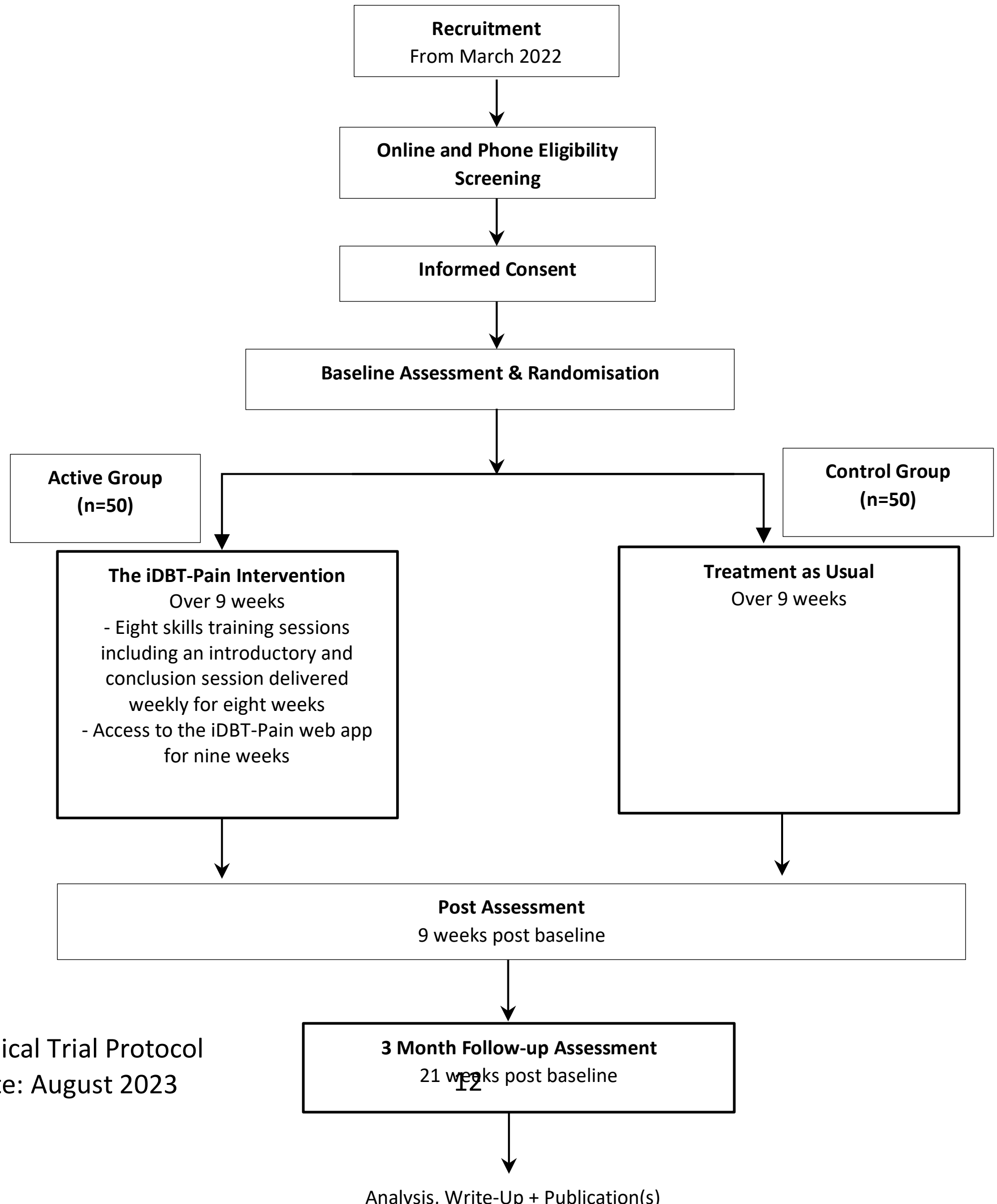

6. Sample Size

The plan is to enrol 100 subjects to complete the trial. The nominated sample is based on a sampled size calculation using SAS 9.4 conducted by biostatistician Dr Nancy Briggs (UNSW Mark Wainwright Analytical Centre). To calculate sample size for the current trial the effect size from our pilot data from the secondary outcome (pain intensity, effect size = .82) (Norman-Nott et al. 2021) has been used as it had a lower effect size than the primary outcome (i.e., emotional dysregulation, effect size = .88). To detect a mean difference of effect size 0.7 between treatment groups in the secondary outcome (i.e., pain intensity), a total of 100 people (50 in each group) will be required. This assumes 80% power, alpha of 0.05 and a within-subjects correlation of 0.2. This aligns with the recommendation for pilot trials in clinical/translational research that states that the number of participants required for a pilot trial is approximately 20-50 per arm (Moore et al., 2011). To account for an expected 15-20% attrition, the total sample size is 100.

7. Selection and Withdrawal of Subjects

Selection

The study will recruit individuals with chronic pain, defined as pain persisting for longer than 3 months. Additional criteria will include:

7.1 Inclusion Criteria

State the inclusion criteria. If there are multiple study groups, define each group in table format criteria or use dot points.

a) Adults aged 18 years or over.
b) Have access to the internet to participant in the Zoom sessions and have access to a smart phone/ tablet device capable of running the iDBT-Pain web app.
c) Commits to fully participate in the skills training e.g., attend all the iDBT-Pain sessions held weekly on Friday lunchtimes (11am to 3pm; AEST) and train skills daily using the iDBT-Pain app.
d) Fluent in speaking and reading English.
e) Average pain rating ≥ 3 for the past seven days (Numeric Rating Scale; NRS).

7.2 Exclusion Criteria

State the exclusion criteria. If there are multiple study groups, define each group in table format criteria or use dot points.

Ensure that the criteria for inclusion and exclusion are provided in lay terminology within the recruitment materials, participant information statement and consent form and the human ethics application.

a) Do not have access to the internet or have no access to a smart device (e.g. phone or tablet) to complete the weekly Zoom sessions and train using the web app.
b) Diagnosed psychotic and personality disorders (e.g., schizophrenia, borderline personality disorder, bipolar disorder, etc.)
c) Uncontrolled mental health disorder.
d) Diagnosis for dementia or Alzheimer's disease.
e) An average pain rating for the past seven days that is less than 3 out of 10 (where 0 is no pain and 10 is extreme pain).
f) Located outside of Australia.

7.3 Recruitment Strategy

Describe the recruitment strategy in detail and explain how this strategy will identify the population studied in this clinical trial.

Established Databases: Primary recruitment methodology will draw on an established patient database available to the research team. This database is a register of individuals with chronic pain who have previously contacted the principal investigator (A/Prof Gustin) and expressed interest in being contacted for potential studies of interest. Eligible participants identified through this regularly updated database will be contacted via email/phone (Attachment 1), and invited to complete an online screening survey (see Section 9.4).

Advertising: We will also recruit participants from within the community using advertisements, including social media (e.g., https://www.facebook.com/groups/australianchronicpainsufferers) and publications specifically targeting individuals with chronic pain. Individuals with chronic pain will be directed to a webpage containing information about the trial, contact details for the research team, (e.g., email; NeuroRecoveryResearch@unsw.edu.au) and a link to the online eligibility screening survey on Qualtrics (Section 9.4) (Attachment 2).

In addition to established recruitment infrastructure, several design characteristics reinforce participant enrolment. First, the online design of the intervention significantly reduces participant travel burden, facilitating access for individuals who may otherwise be unable to attend multiple skills training sessions. This design has been very well received by participants in our pilot investigation (see Norman-Nott et al., 2021). Further, the online design contributes to the ecological validity of the intervention as participants can continue their practice in the environment where they have learnt the skills using the self-paced iDBT-Pain app. We will facilitate participants' transportation for MRI assessments as needed. Additionally, it is important to note that the current study does not draw from an acute patient population but rather individuals who have chronic pain lasting upwards of three months. As no limit is placed on type of chronic pain, this likewise increases enrolment opportunity.

7.4 Screening

Describe the screening process and explain how the research team will inform participants who cannot participate in the trial.

The screening process is divided into two steps, (1) online screening, and (2) phone screening. The script for participant screening is in Attachment 3.

Online Screening Survey: To determine study eligibility, online screening will be used for all applicants using an online survey administered through the Qualtrics. The online screening survey will ask questions about age and demographics, chronic pain condition, existing and previous psychiatric conditions, MRI contraindicator's, current medications, internet accessibility, fluency in reading and speaking in English, contact details, and location (approx. 10 min).

Eligible applicants will then be contacted by email to arrange a time for a screening interview by phone to determine eligibility (see Phone Screening below). Non-eligible participants will receive an email letting them know they are not eligible to participate and informing them that their data will be deleted from the files associated with this trial.

As part of the online screening, to determine MRI eligibility, participants will be asked if they would like to participant in an MRI. If participants answer yes, they will be asked to complete a safety checklist (standard MRI checklist) that is designed to identify metal in their body that could pose a hazard during Magnetic Resonance Imaging (MRI). Participants with MRI contraindications (e.g., brain aneurysm clip, neural stimulator, cardiac pacemaker, cochlear implant, insulin pump or metal shrapnel/bullet) may be excluded. Contraindications will be assessed using standard institutional measures (provided by the NeuRA MRI facility). All eligibility screenings will be reviewed by the NeuRA radiographer (see MRI session description below). Participants who are ineligible for an MRI scan can still participate in all other study elements. Imaging will only be offered to participants in the treatment conditions.

Phone Screening: Applicants who are eligible based on the online screening will be contacted by a member of the research team to collect more information about the individuals' current circumstances and areas of concern and provide information about the skills training (approx. 15 mins).

Individuals who are ineligible to participate in the trial will be sent an email, notifying them that they are ineligible and thanking them for their interest in our research (see email script below):

Email script to notify of ineligibility:

> Thank you for your interest in our trial: IDBT-Pain - Internet Delivered Skills Training for Chronic Pain (iDBT-Pain). We had some very strict criteria for inclusion in this trial and unfortunately you are not eligible to participate. We understand this may be disappointing for you and we are very sorry that you cannot be a part of the trial.
>
> However, if you agreed to have your details kept on record when you completed the online form, we will retain your information on our secure database and will contact you about future research. If you have not indicated that you agree to have your contact details kept on file, we will delete all your details from our files.
>
> Further, if you are interested in participating in trials in the future, please visit [website address TBC] where we regularly share the details of our upcoming research.
>
> Thank you again for your interest
>
> Kind regards

Applicants that screen eligible will be contacted by email or phone to advise them of their eligibility and will be emailed the Participant Information Statement and Consent Form to review. In this communication applicants will be asked to have a call with a member of the research team in approximately three days' time to discuss the consent process. Eligible participants will be given the opportunity to not progress if they are no longer interested in the trial.

7.5 Consent

Describe the process for collecting consent.

Informed Consent: Informed consent will be completed after participants have screened eligible, stated their interest and before they complete the baseline questionnaires. The Participant Information Statement and Consent Form (Attachment 4) will include a description of the study, including that they will be randomised to one of two conditions (treatment or treatment-as-usual) and a description of the skills training intervention. Procedures covered for the Informed consent will include (a) completion of questionnaires (Attachment 5) at 3 timepoints – Pre-intervention (Baseline assessment); post intervention (post-assessment); and 3 months post intervention respectively (follow-up); (b) participation in the iDBT-Pain sessions and skills training using the iDBT-Pain app; (c) if eligible, and interested, complete brain imaging at 2 timepoints – baseline assessment and post-assessment; (d) consent to video recording of the iDBT-Pain sessions on Zoom.

Informed consent will be provided through a secure online application (Qualtrics) but will be reviewed by phone with the participants to ensure understanding. The opportunity for participants to ask questions is emphasized during this process – both during consent, and throughout the entire study course. In order to ensure that participant questions are addressed in thorough fashion, a teach-back method of informed consent will be used to ensure the participant fully understands participation. In this process, the person obtaining consent asks the participant to restate information in their own words. Participants must be able to demonstrate understanding of the study through conversation with the research staff. Consent will be treated as an ongoing process – willingness to continue with the research is assessed at each contact through conversation.

7.6 Withdrawal of Consent or Participant

Describe the subject withdrawal process and describe the procedures to ensure participants safety is monitored throughout the withdrawal process.
Provide detailed procedures for informing participants of the risks and the ongoing safety monitoring required in circumstances when terminating the trial interventions, including:

- When and how to withdraw subjects from the trial, the trial interventions, investigational product treatments, investigational device treatment or health, psychiatric, physiological, or psychological treatments.
- The type and timing of the data to be collected for withdrawn subjects.
- Whether and how subjects are to be replaced
- The follow-up for subjects withdrawn from investigational product treatment/trial treatment.

Participant Withdrawal: In the PISCF, participants will be advised of both the potential risks and benefits from participating in the iDBT-Pain trial. Participants will have the ability to communicate with the research staff throughout the study and will be advised to contact them if they have concerns resulting from participation in the skills training or assessments or if they may be considering withdrawing from the study.

Participants may withdraw at any time. Participants wishing to withdraw can do so by completing the 'Withdrawal of Consent' form, which is accessible online via contact with iDBT-Pain team and also provided at the end of the Participant Information Statement and Consent Form; or they can email (e.g. neurorecoveryresearch@unsw.edu.au) or phone (trial phone number to be confirmed) the research team and tell them they no longer want to participate. Should participants decline to complete the withdrawal form, research assistants will withdraw participants once intention to withdraw is expressed. Their withdrawal will not affect their relationship with UNSW or research team members.

If participants do withdraw from the research study, the researchers will not collect additional information from them. Participants can request that any identifiable information about them be withdrawn from the research project.

8. Treatment of Subjects

Please provide a detailed description of the trial participants' trial procedures during their involvement in the trial.

Recruitment and Screening Procedure: As outlined in section 3.9, recruitment methodology will primarily draw on an established database of people with chronic pain who will be emailed or phoned by a member of the research team about the trial. The secondary recruitment strategy is community advertisements (e.g., social media and relevant publications). Those individuals interested in participating will be directed to a webpage containing information about the trial, contact details for the research team, (e.g., email; NeuroRecoveryResearch@unsw.edu.au) and to start the screening process if they choose. The screening process has two steps (see Section 9.4) and encompasses an online screening survey accessible on the study webpage (step 1), and a screening phone call (step 2) to determine eligibility for the study. The full screening process is predicted to take no longer than 30 minutes and it is anticipated it will not cause any harm or discomfort than would typically be experienced in everyday life. As part of the online screening, to determine MRI eligibility, participants will also be asked to complete a safety checklist (standard MRI checklist) (Attachment 7) that is designed to identify metal in their body that could pose a hazard during Magnetic Resonance Imaging (MRI). Participants who are ineligible for an MRI scan can still participate in all other study elements. Inclusion and exclusion criteria used to determine eligibility are stated in section 9.1 and 9.2. During the screening process potential participants will have the opportunity to ask questions about the study.

Consent Procedure: Individuals that are eligible to participate in the study will be invited to complete the informed consent process detailed in section 9.5, whereby they will be provided with a copy of the PISCF by email, asked to review it and will have the opportunity to ask a member of the research team questions via phone and email. They will then be invited to electronically sign the PISCF through a secure online application (Qualtrics) (see Section 9.5). Participants will be advised that they can withdraw at any point and no further data will be collected (see Section 9.6).

Randomisation Procedure: Forty-eight participants will be recruited to the study (see 6. Sample Size). Following informed consent, and before baseline assessment participants will be randomly allocated to groups (treatment or treatment-as-usual) in blocks of 6. A randomisation

schedule will be created in Excel by the statistician who is not involved in the recruitment, treatment, or data collection to minimise risk of bias.

Allocation of participants to randomisation schedule: Participants will have been assigned a participant ID in Qualtrics during the study screening process (see Section 7.4). This ID will be provided to a member of the research team to be added in the next available cell in the randomisation schedule Excel sheet. The member of the research team that adds the participant ID to randomisation schedule Excel sheet will not have access to any information about the participant except the participant ID and will not be involved in the recruitment, treatment, or data collection. Once the participant has been added to the Excel sheet, the team member will advise the skills trainer of the participants group allocation and the group allocation will be updated by the skills trainer in Qualtrics. The skills trainer will communicate the group allocation to each participant by phone or email.

Skills Training: Participants in the treatment condition will be advised that they will complete the emotional recovery skills training over nine weeks (iDBT-Pain intervention) in groups of up to 12 participants. Participants will be advised that the iDBT-Pain skills training involves a total of eight Zoom sessions (including an intro and concluding session), approximately every 7 days delivered over eight weeks. These participants will be advised that for these eight weeks, and for one week after the concluding session, they will have continued access to a web app accessed via their own or, as needed, a supplied smart device. Following the study, participants will have ongoing access to the web app on their personal devices.

Each participant in the skills training condition will be provided with worksheets informed by the DBT Skills Training Manual (Linehan, 2015), that will be referred to during the Zoom sessions and will facilitate homework.

Treatment-as-usual: Participants in the treatment-as-usual condition may receive treatment options that can be any of those offered by the healthcare professionals they would normally choose to consult with in the community. In other words, the participants in the treatment-as-usual condition will be advised they will not receive treatment determined by the study or funded by it. It will be explained to the participants that the role of being in this group is very important to the study because the outcomes gained by this group set the bar to know whether the intervention (iDBT-Pain) is any better or not. It will further be explained to these participants that they will have access, at the study conclusion (e.g., following the 3-month follow-up assessment), to the iDBT-Pain web app accessible via their own smart device.

Data Collection Procedure: Assessment times for both the treatment and treatment-as-usual conditions will be at baseline, post-intervention (9-weeks post baseline assessment) and at 3 months following the post-intervention assessment. Baseline assessment will begin once 24 participants are in a group and randomised. Assessments will encompass self-report questionnaires (see section 10.1), and eligible participants will be invited to undertake MRI scanning. Questionnaires will be completed online via Qualtrics survey platform and will take a maximum of 90 minutes to complete. MRI scanning will be in-person at NeuRA, Sydney (see section 10.1). Participants will be advised that if they cannot or choose not to participate in the MRI they may still participant in all other aspects of the study. Additionally, post-intervention, qualitative data will be collected from participants in the treatment condition via a semi structured interview with a member of the research team via Zoom or phone call.

- *Data Storage Procedure*: Data safety will comply with the National Statement on Ethical Conduct in Human Research. Only the IDBT-Pain research staff who interact with participants will see identified data'. Qualtrics is a data capture software that ensures curation of data via the use of standardised data collection. Its electronic capture and pre-defined fields ensure data consistency and integrity. Qualtrics uses Transport Layer Security (TLS) encryption (also known as HTTPS) for all transmitted data. The data is stored on a secure server that is hosted in a trusted data centre in Sydney (the data centres are independently audited using the industry standard SSAE-18 method). The server is protected by high-end firewall systems. Backups are performed daily. Qualtrics is recommend by UNSW for sensitive data as captured in this trial, and UNSW holds an institutional license for Qualtrics. Qualtrics is password protected via zID and password.

Once data is exported out of Qualtrics, for example into Excel or SPSS for data analysis, it will be stored in de-identified format on OneDrive/ Teams on the UNSW servers with a unique participant identification code. During the screening process (see section 7.4) participants will have been automatically assigned a participant ID in Qualtrics. A master file containing participants identifiable information (i.e. name and email address) and corresponding participant identification code will be stored separately in an Excel sheet on the UNSW servers accessible to research staff via password protection.

De-identified imaging data will be stored on a password protected UNSW secure server, with access restricted to the iDBT-Pain research team as nominated by the coordinating principal investigator. These data units are not associated with any participant identifying information. Hard copy data will be stored securely within locked filing cabinets in a locked office in the Biolink Building, School of Psychology, UNSW. It will be kept in separate files/cabinets to those containing participant details and trial identification numbers. Only approved iDBT-Pain researchers will have access to this information. If problems with data consistency are identified, audits will be conducted at the advice of the Trial Management Group.

The iDBT-Pain sessions, delivered by a DBT skills trainer via Zoom in groups of up to 12 participants, may be recorded and if so, will be saved on a password protected server at UNSW. Recorded sessions will be only available for the researchers, and may be sent to participants, from this trial only, in the case that a session is missed and the participant needs to catch up on content. This is described to participants in the PISCF.

Cybersecurity: To ensure participant cybersecurity and prevent potential hacking into the Zoom sessions, access to the Zoom sessions will be password protected. Moreover, the waiting room function within Zoom will be utilised meaning that the skills trainer will need to give each participant access to the session further securing against hacking.

Prize Draw: Participants will be advised that they can choose to enter a prize draw to receive one of two Westfield $200 gift cards. To notify us of their request to be entered into the draw, participants must check a box to consent to be entered into the draw when completing the informed consent form.

The prize draw is a token appreciation to thank participants for the time taken to participate in the study. The draw will be conducted following the completion of the 3-month assessment.

Participants that do not complete the study will not be eligible for entry into the draw. Gift cards will be posted to the two participants that win the prize draw.

10.1 Trial Intervention

- Describe the trial interventions, specify the qualifications and experience required by personnel delegated these responsibilities.

Trial Intervention: iDBT-Pain is an online skills training encompassing a total of eight 60-90-minute-long sessions (including an introductory and concluding session), delivered over the internet using Zoom. These sessions will be delivered as part of a group of around 8 to 12 participants and will occur on a Friday lunchtime sometime between 12pm and 2pm AEST approximately every 7 days across eight weeks. For the duration of the eight weeks, and for one week after the concluding Zoom session, participants will have continued access to a web app (iDBT-Pain web app) which participants will access on their own smart device (e.g., iPhone or computer) with their own secure password and username. The iDBT-Pain app includes videos and tasks to encourage skills practice between the Zoom sessions. This intervention is designed to teach DBT skills in mindfulness, emotion regulation, and distress tolerance alongside psychoeducation about chronic pain. Each of the eight session builds on the content of the last to give participants a complete set of skills. This format for the skills training mirrors that used in our successful pilot study where no difficulties in adherence were observed. Nevertheless, to facilitate the completion of all skills by all participants, the iDBT-Pain sessions will be recorded via Zoom. Thus, in the instance that a participant in unable to attend one of the iDBT-Pain sessions they will be sent the session as a recording which they can watch at the earliest opportunity to catch up on the content before the next session. The iDBT-Pain skills training in the current study will follow the study protocol developed and trialled by Norman-Nott et al (2021) and will follow the below session outline:

Session	Module	Skills	Function
**Intro**<br><br>**Week 1**	Introductory Session	Using Zoom for the iDBT-Pain sessions<br>Accessing the iDBT-Pain app	Introduce the tools associated with the intervention<br>Introduce the group to one another
**1**<br><br>**Week 2**	Mindfulness	Observing, describing, participating	The core skills of mindfulness will be introduced for nonjudgemental awareness of the present.
	Chronic Pain Psychoeducation	Understanding the association between pain, emotions, and the brain.<br>How to calm down the overactive brain.	Teach the theory behind the relationship between chronic pain and increasing emotional issues<br>Explain the purpose for mindfulness for the overactive brain
**2**<br><br>**Week 3**	Mindfulness	Nonjudgmentally, One-mindfully, Effectively	Introduce further mindfulness skills in order that participants practice mindfulness with skilful effectiveness

	Chronic Pain Psychoeducation	Understanding the association between pain, negative emotions, and the brain<br>How to calm down the overactive brain.	Recap session 1 learnings and align with the new mindfulness skills for session 2
**3**<br><br>**Week 4**	Emotion Regulation	Understanding Emotions	Teach how to identify and label emotions as a function for emotion regulation
	Chronic Pain Psychoeducation	The relationship between emotion dysregulation and chronic pain	Explain the need for emotion regulation skills in the context of chronic pain
**4**<br><br>**Week 5**	Emotion Regulation	Handling Unwanted Emotions	Teach skills to reduce the frequency and quantity of unwanted emotions
	Chronic Pain Psychoeducation	The relationship between emotion dysregulation and chronic pain	Recap session 3 learnings and align with new emotion regulation skills
**5**<br><br>**Week 6**	Emotion Regulation	Building Mastery and Cope Ahead	Teach skills to build future resilience against intense emotion
**6**<br><br>**Week 7**	Distress Tolerance	TIP (Temperature of face, intense exercise, paced breathing), Distract	Teach skills that help weather crises and intense negative emotions
**Conc**<br><br>**Week 8**	Concluding Session	Mindfulness, emotion regulation and distress tolerance	Recap the skills learnt and the association between chronic pain and difficulties with emotions

Skills Trainer: The iDBT-Pain skills training will be administered by a DBT skills trainer (Nell Norman-Nott) who has completed the DBT Foundational Program and DBT Skills Training Program run by Psychwire, Behavioral Tech and Marsha Lineham (founder of DBT). Given that the iDBT-Pain skills training is designed to teach new capabilities in pain and emotion management and is not psychotherapy, having this program run by a DBT skills trainer is sufficient to teach such required skills. It is not anticipated that participants will experience substantial distress associated with this intervention because it is a skills training designed to teach new capabilities in pain and emotion management not psychotherapy. Nevertheless, A/Prof Gustin is an AHPRA registered psychologist and can be contacted if any unforeseen distress arises as a result of the skills training. If for any reason participants do experience increased distress, they will be able to discontinue the program, and after a discussion with the skills trainer (who will consult with the principal investigator), be referred to alternative sources of support. Participants will never be required to practice or learn skills more than they would like to, and the skills trainer will not coerce or persuade the individual to keep going with the program if the participant wishes to discontinue.

Aside from the delivery of the skills training, investigators involved in this trial are experienced in data collection, brain imaging and have a track record of leading rigorous RCTs. Detailed protocols are peer-reviewed and published prior to commencement (e.g., BMJ open, Norman-Nott et al., 2021), and the resultant papers are published in the top medical journals (e.g., Lancet, Hancock et al., 2007; Lancet Neurology, Pain, Gustin et al., 2010).

- Describe all trial treatments, measures, and procedures that participants will complete or used to collect trial data and detail the instructions for administering these procedures.

Questionnaires: The primary outcome in this trial is emotion dysregulation which will be measured via self-report using the 18-item version of the Difficulties in Emotional Regulation Scale (DERS-18).

The secondary outcomes include:

- Numeric Rating Scale (NRS) to measure pain intensity.
- NIH Toolbox - Perceived Stress Survey-10 (PSS-10) to measure perceived levels of stress
- Beck Depression Inventory (BDI) to measure symptoms of depression
- State-Trait Anxiety Inventory (STAI) subscale for state anxiety to measure symptoms of anxiety
- Medical Outcomes Study – Sleep Scale (MOS-SS) to measure sleep quality.
- Temperament and Character Inventory Harm Avoidance Scale (TCI-HA) to measure the character trait of harm avoidance.
- COMPAS-W Scale of Wellbeing (COMPAS-W) to measure wellbeing.
- Dialectical Behaviour Therapy Ways of Coping Checklist (DBT-WCCL) to measure emotional effectiveness and coping behaviours.
- Healthcare Utilisation questionnaire (HUQ) to measure usual healthcare treatment.
- Health-Related Quality of Life Scale (EQ-5D-5L) to measure quality of life for economic analysis
- Satisfaction with Life Scale (SWLS) to measure life satisfaction
- PROMIS Pain interference instrument-8 (PII-8) to measure pain interference
- Patient Global Impression of Change Scale (PGIC) impression of change following the intervention.
- Usefulness, Satisfaction and Ease of Use Scale (USE) to measure the satisfaction and useability of the intervention.
- Chronic Pain Medication and Health Intervention Questionnaire (CPM-HIQ) to measure current and prior medication and health interventions
- The Awareness of Social Inference Test-Short (TASIT-S) to measure social cognitive skills

The table below details the versions of the questionnaires to be administered, the conditions, assessment times, and approximate time taken to complete:

	Condition	*Included in Attachments*	*Baseline Assessment*	*Post-Assessment*	*3-month follow-up Assessment*
DERS-18	T & TAU	√	X	X	X
NRS	T & TAU	√	X	X	X
PROMIS-PI	T & TAU	√	X	X	X
PTSD-C	T & TAU	√	X	X	X
BDI	T & TAU	√	X	X	X
STAI	T & TAU	√	X	X	X
NIH-PSS	T & TAU	√	X	X	X

MOS-SS	T & TAU	√	X	X	X
TCI-HA	T & TAU	√	X	X	X
COMPAS-W	T & TAU	√	X	X	X
DBT-WCCL	T & TAU	√	X	X	X
SWLS	T & TAU	√	X	X	X
EQ-5D-5L	T & TAU	√	X	X	X
HUQ	T & TAU	√	X	X	X
CPM-HIQ	T & TAU	√	X	X	X
USE	T	√		X	X
PGIC	T & TAU	√		X	X
TASIT-S	T & TAU		X	X	X
Duration			80 min	90 min	90 min

T = Treatment Condition, TAU = Treatment-as-usual Condition, DERS-18= Difficulties in Emotion Regulation Scale-18, NRS = Numeric Rating Scale, PROMIS-PI = PROMIS Pain Interference Instrument, PTSD-C = PTSD Checklist – Civilian version, BDI = Beck Depression Inventory, STAI = State-Trait Anxiety Inventory, NIH-PSS- = NIH Toolbox Perceived Stress Survey, MOS-SS = Medical Outcomes Study Sleep Scale, TCI-HA = Temperament and Character Inventory Harm Avoidance Scale, COMPAS-W = COMPAS-W Scale of Wellbeing, DBT-WCCL = Dialectical Behaviour Therapy Ways of Coping Checklist, SWLS = Satisfaction with Life Scale; EQ-5D-5L = Health-Related Quality of Life Scale, HUQ = Health Utilisation Questionnaire, USE = Usefulness, Satisfaction and Ease of Use Scale, CPM-HIQ = Chronic Pain Medication and Health Intervention Questionnaire, PGIC = Patient Global Impression of Change, TASIT-S = The Awareness of Social Inference Test-Short

At baseline assessment, the questionnaires will be self-reported by participants sequentially online via Qualtrics and will be completed in the same manner post-intervention (i.e., 9 weeks after baseline and at 3 months follow-up). The full questionnaires can be seen in Attachment 5

Brain Imaging: Participants in the skills training group who are eligible and interested will attend the MRI facility at NeuRA for imaging assessment at baseline and post-intervention. The MRI equipment has been in routine clinical use for over two decades and is approved by the Australian Therapeutic Goods Administration. Each MRI session (pre-intervention, and post-intervention) will be an identical 60-minute multimodal scan procedure conducted with a Philips Ingenia 3T MRI scanner. Prior to scanning, per safety policy at NeuRA, all participants will complete an MRI safety form to probe for metal in the body or other contraindications. This form will be reviewed by a radiographer before a participant is allowed to enter the scanner room. Structural, functional and biochemical neuroimaging data will be collected.

Semi-Structured Interview: Participants will be asked by a member of the research team questions about the iDBT-Pain skills training to attain qualitative data about areas that are effective and aspects that could be improved (Attachment 8). The interview will be conducted by a member of the research team with each participant individually by telephone or via Zoom. The interview will take approximately 20-minutes and will be conducted at a time convenient to the participant. The participants responses will be entered by the researcher directly into a form in Qualtrics and will be stored in accordance with the steps detailed in Section 10. The semi-structured interview will not be recorded.

- Specify how and when the intervention will be administered to trial participants. Indicate the names of the products to be used and provide the instructions for administration.

The iDBT-Pain skills training encompasses the iDBT-Pain sessions administered online via Zoom and the iDBT-Pain web application accessed via smart device (e.g., smart phone, tablet, computer). Participants in the treatment group will receive the intervention when 24 participants have been recruited and randomised and after baseline assessment. The administration will adhere to that of our previous trial (Norman-Not et al, 2021).

- List the medication(s)/treatment(s) permitted (including rescue medication) and not permitted before or during the trial.

No medications will be administered as part of this trial because the intervention is a skills training. Participant's existing medication (e.g., pain analgesics) will be documented at pre and at post-assessment. Participants will be asked to, where possible, refrain from making changes to their medication throughout the trial and will be asked to advise the research team if they do make changes to their medication during the trial.

- Describe the follow-up period(s) for subjects for each type of intervention.

Follow-up periods for participants in both the treatment and the treatment-as-usual conditions will be as follows:

Baseline assessment: Participants in both conditions will complete online self-report assessments via the online Qualtrics survey platform to measure emotion dysregulation, social cognition, pain intensity, depression, anxiety, stress, sleep quality, harm avoidance, emotional effectiveness, coping behaviours, healthcare treatment, and quality of life.

Post-assessment: At 9-weeks post baseline assessment, the self-report measures administered during the baseline assessment will once again be completed by participants in both conditions. In addition, participants in the treatment condition will self-report their satisfaction with, and the useability of, the iDBT-Pain intervention and will complete a semi structured interview with a member of the research team. Self-report measures will be completed online via Qualtrics and the semi-structured interview will be via phone or Zoom at a time convenient for the participant.

Follow-up assessment – 3 months: At 3 months following the post-assessment the self-report measures administered during the baseline assessment will once again be completed by participants in both conditions.

- Specify any differences between the trial intervention, treatment, placebo arms for the trial.

Treatment Condition: Participants in the treatment condition will receive the iDBT-Pain intervention. The iDBT-Pain intervention involves one 60-90 minute introductory session, six 60–90 minute iDBT-Pain sessions delivered by a DBT skills trainer via Zoom in groups of 8 to 12 participants, and one concluding session delivered weekly (every 7 days) over eight weeks. As part of the iDBT-Pain intervention, for the duration of the eight weeks, and for one week after the concluding Zoom session, participants will have continued access to a web app (iDBT-Pain web app) which participants will access on their own smart device (e.g., iPhone or computer) with their own secure password and username. The iDBT-Pain app includes videos and tasks to encourage skills practice between the Zoom sessions and to maintain the skills in the week

following the concluding session. Participants will be provided with a set of clear instructions to set up the app and to access the sessions (Attachment 9).

Fidelity and Consistency Across Treatment Groups: Participants in the treatment condition will be in one of two skills training groups consisting of 12 participants for their iDBT-Pain sessions. The structured protocol (see section 10.1) for the iDBT-Pain sessions will ensure treatment fidelity and consistency across the two groups to mitigate effects of confounding factors that may arise due to different groupings.

Treatment-as-usual condition: In contrast to the treatment condition, the treatment-as-usual condition will receive no intervention as part of the trial. Participants in the treatment-as-usual condition may receive treatment options as offered by the healthcare professionals they would normally consult with in the community (see Section 10).

Upon conclusion of the study (e.g, following 3-month assessment) all participants will retain (if in the treatment group) or receive (if in the treatment-as-usual group) access to the iDBT-Pain app for personal use on their own smart device.

9. Safety and Monitoring

11.1 Assessment of Safety Event Report Forms

Safety reports will be assessed on the seriousness, causality, and expectedness of the event to the trial treatment(s), intervention(s), investigational medical product(s), investigational medical device(s). The following are known and expected adverse effects, harms, risks or discomforts associated with trial procedures, treatments or interventions.

a) Known Adverse Effects

Provide a detailed list of all adverse effects of the trial intervention that participants may (or have the potential) experience.

DBT skills training is an evidenced-based protocol for emotion dysregulation (Linehan, 2015; Neacsiu et al., 2014). In our pilot trial there were no adverse events (Norman-Nott et al., 2021), and prior research further shows that DBT skills training is a standardised approach with no known side effects reported (Linton, 2010; Sysko et al., 2016). Thus, we do not anticipate any study related adverse events in this current trial.

b) Known Harms, Risks or Discomforts

Provide a detailed list of all adverse effects of the trial treatments that participants may (or have the potential) experience.

Psychological Risks (mild and minimal likelihood): The aim of the iDBT-Pain skills training is to enable participants to better appraise and express emotions which are impacted by the effect of chronic pain on their lives. On exposure to this information, a very small minority of participants may experience temporary and transient increases in distress. However, this was not observed in our previous study (Norman-Nott et al., 2021). We will also minimise this risk by excluding people with diagnosed psychiatric conditions or mental health disorders (see Exclusion Criteria). If for any reason participants do experience increased distress, they will be able to discontinue the program, and after a discussion with the skills trainer (who will consult with the principal investigator), be referred to alternative sources of support. Participants will never be required to

practice or learn skills more than they would like to, and the skills trainer will not coerce or persuade the individual to keep going with program if the participant wishes to discontinue.

There are no anticipated risks associated with conducting the skills training online or with using Zoom as a tool for this purpose. It is anticipated that by having the skills training online it is an advantage for this population who may struggle with mobility due to chronic pain symptoms making access to a face-to-face session harder than online. To ensure that participants feel comfortable using Zoom and do not experience distress using this platform, they will be taken through a short training on this tool (10 mins) during the introductory session (see Section 10.1). For technical issues with connecting to Zoom, the participants will be advised that they can contact the researchers (e.g., by emailing NeuroRecoveryResearch@unsw.edu.au).

Physical Risks (mild and minimal likelihood): There may be some discomfort in terms of fatigue by attending up to nine hours of Zoom sessions. To mitigate this, sessions will be spaced evenly throughout the intervention period with one per week over eight weeks (see Section 10.1), with sufficient time between sessions and breaks as necessary. Participants may similarly experience some fatigue and boredom when completing the survey and questionnaires. To mitigate this where possible the short forms of the questionnaires have been selected (e.g., DERs-18).

MRI risks (mild to minimal likelihood): MRI is a non-invasive and painless procedure. The MRI equipment has been in routine clinical use for over two decades and is approved by the Australian Therapeutic Goods Administration, the European Union, and the USA Food and Drug Administration. During the MRI scanning, participants may become anxious or claustrophobic. If for any reason a participant becomes uneasy or uncomfortable, they may stop the procedure at any time; a buzzer will be given to hold for this purpose. The scanner makes loud banging noises during the procedure, so earplugs and headphones will be provided to minimise the noise.

There are no risks associated with the MRI process, and there are no short- or long-term side effects. There is a low possibility of nerve stimulation which may manifest as a tingling sensation or twitching; however, this will not be a risk to the participants' health.

Participants will not be allowed to have an MRI examination, if they have any of the following conditions: a cardiac pacemaker; metal fragments in eyes, skin, body; heart valve replacement; brain clips; venous umbrella; history of being a sheet metal worker or welder; aneurysm surgery, intracranial bypass, renal, aortic clips; prosthetic devices such as middle ear, eye, joint or penile implants, joint replacements; hearing aid; neurostimulator; insulin pump; intrauterine device (IUD); shunts/stents; metal mesh/coil implants; metal plate/pin/screw/wire, or any other metal implants; permanent eyeliner, eyebrows. The participants will be asked to fill in an MRI safety form before entering the scanning room. A trained MRI radiographer will be present throughout the whole procedure, monitoring the participant's scanning.

Cerebral MRI is most commonly used in diagnosing neurological diseases, and rarely may find unexpected pathology. In that event, a neurologist from NeuRA's imaging facility, who will look at all structural images, will release the report to the researcher in charge of this study (A/Prof. Sylvia Gustin) who would then be responsible for any follow up. If abnormalities should be found, A/Prof. Sylvia Gustin would be responsible for advising the participant of the abnormal report and liaising with the participant for the submission of the report to their primary health care provider.

Participants may feel uncomfortable, and/or experience claustrophobia being in the MRI, but we will try to minimise that discomfort by informing them of

the testing environment ahead of time; informing them they can pause or discontinue at any time, and providing noise reducing earplugs.

Adverse event and serious adverse event reporting

DBT skills training is an evidenced-based protocol for emotion dysregulation (Linehan, 2015; Neacsiu et al., 2014), and research shows that DBT skills training is a standardised approach with no known side effects reported (Linton, 2010; Sysko et al., 2016). Thus, we do not anticipate any study related adverse events in this current trial.

Nevertheless, if any adverse events do occur, the IDBT-Pain research team will collect data, document the information and report to the relevant bodies within the timeline as required by the UNSW HREC (detailed below).

Participants will have the ability to communicate with the research staff throughout the study, via email (neurorecoveryresearch@unsw.edu.au) and phone (trial phone number to be confirmed) and can contact them if they have concerns that may be related to, or resulting from any aspect of the trial. If necessary, the participant will also be advised to seek care from their usual health care professional. We will further provide (if necessary) a list of counselling services participants may access (e.g. Beyond Blue, Lifeline etc) (Attachment 10).

Adverse event data will be collected via participant text, phone, or email contact with the IDBT-Pain research team, and the research team will monitor any difficulties participants may be having during the skills training.

For all adverse events an evaluation form will be completed that will include a description of the event, a classification of seriousness, assessment of potential relationship to the intervention, assessment of need for change in the consent or the study activities, a summary of known prior health issues, and event outcome.

11.2 Adverse Events or Adverse Reactions

Adverse events (AE) are considered any untoward medical occurrence in a patient or clinical trial participant administered the intervention, which does not necessarily have a causal relationship with this treatment.
Adverse Reactions (AR) are considered untoward and unintended responses to the trial intervention related to any intervention procedures.

AEs and ARs are assessed using the safety monitoring flow chart. Those classified as "not serious" are assessed by the qualified physician/medical expert specified in section 2 of the protocol. The Qualified Physician cannot delegate this responsibility to other research personnel.

Adverse event reports must be reported to the Principal Investigator within 24 hours of being notified by the participant. All adverse event reports must be recorded in the UNSW Safety Monitoring Register Template.

11.3 Serious Adverse Events

Serious Adverse Events (SAEs) that result in or lead to one or more of the following and the event is **not related** to the trial intervention:

- The death of a trial participant.

- A life-threatening illness or injury involving a trial participant.
- A participant's permanent impairment of body structure or body function.
- In-patient or prolonged hospitalisation (not for a pre-existing condition or an elective surgery) of a trial participant.
- Medical or surgical intervention to prevent life-threatening illness or injury or permanent impairment to a body structure or function of a trial participant.
- Fetal distress, fetal death or congenital abnormality or birth defect.

SAE reports are classified following the safety assessment flowchart and are assessed by Sponsors Independent Medical specified in section 2 of the protocol. The Sponsors Independent Medical cannot delegate this responsibility to other research personnel. SAE reports are reported to the Coordinating Principal Investigator immediately or within 24 hrs of the event occurring. SAR reports must be recorded in the UNSW Safety Monitoring Register Template.

11.4 Serious Adverse Reactions

A Serious Adverse Reactions (SAR) is an SAE that is **related** to the trial intervention. SAR reports are classified following the safety assessment flowchart and are assessed by Sponsors Independent Medical expert specified in section 2 of the protocol. The sponsors independent medical expert must determine whether the SAR was expected or unexpected. The Sponsors Independent Medical cannot delegate this responsibility to other research personnel.

a) Expected Serious Adverse Reaction

A serious adverse reaction by its nature, incidence, severity, or outcome is anticipated and identified in the current version of the intervention safety information is classified as a SAR report. SAR reports are reported to the Coordinating Principal Investigator, immediately or within 24 hrs of the event occurring. Serious Adverse Reaction reports must be recorded in the UNSW Safety Monitoring Register Template.

b) Suspected Unexpected Serious Adverse Reaction (SUSAR)

A serious adverse reaction by its nature, incidence, severity, or outcome is unanticipated and not identified in the intervention's instructions for use or safety information is classified as a SUSAR.

Fatal or life-threatening Australian SUSAR reports are reported to the Coordinating Principal Investigator, the sponsor's delegate and the approving HREC within 7 calendar days after being made aware of the case follow up information reported within a further 8 calendar days.

All other Australian SUSAR reports are to be reported to the Coordinating Principal Investigator, the sponsor's delegate and the approving HREC within 15 calendar days after being made aware of the case follow up information reported within a further 8 calendar days. SUSAR reports must be recorded in the UNSW Safety Monitoring Register Template.

11.5 Significant Safety Issue (SSI)

A safety issue that could adversely affect participants' safety or materially impact the trial's continued ethical acceptability or conduct. The Human Research Ethics Committee and Sponsor's Delegate must be notified of all significant safety issues within 15 calendar days of the sponsor instigating or being made aware of the issue. SSI reports must be recorded in the UNSW Safety Monitoring Register Template.

11.6 Urgent Safety Measure (USM)

A measure that is taken to eliminate an immediate hazard to a participant's health or safety. Significant safety issues where an urgent safety measure is required to be taken to eliminate an immediate hazard must be classified as a significant safety issue requiring an urgent safety measure. The Human Research Ethics Committee and the Sponsor's Delegate must be notified of any significant safety issues that meet the definition of an urgent safety measure should be notified within 72 hours. Examples include:

- a serious adverse event that could be associated with the trial procedures and that requires modification of the conduct of the trial
- a patient population hazard, such as lack of efficacy of an intervention used for the treatment of a life-threatening disease.

USM reports must be recorded in the UNSW Safety Monitoring Register Template.

11.7 Safety Assessment Flow Chart Investigational Medical Product Trials

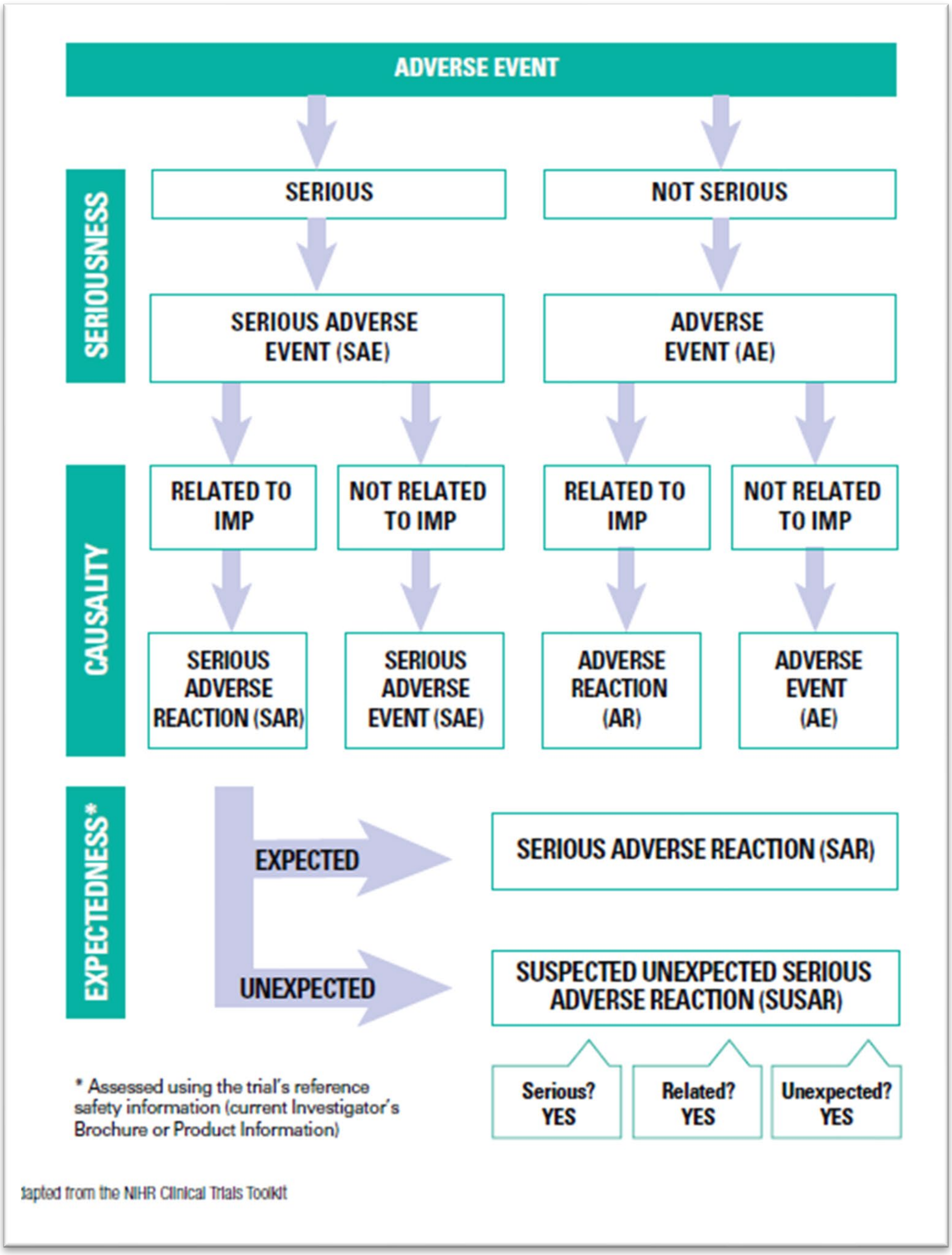

11.8 Register of Clinical Trial Safety Monitoring Reports

A register of all event reports assessed and classified is to be retained by the Coordinating Principal Investigator and reported to the trial sponsor annually and the HREC if required.

11.9 Reporting of Clinical Trial Safety Monitoring Reports

Single case reports of Adverse Events Adverse Reactions, Serious Adverse Events (SAEs), Serious Adverse Reactions (SARs), reports do not need to be reported to the UNSW Sponsor's Delegate or the HREC. All single case reports must be recorded in a safety monitoring register and are reported to the UNSW Sponsor's Delegate annually.

a) Emerging Safety Issues

The Trial Management Group (TMG) will oversee the day-to-day conduct of the trial. They will closely review all aspects of the conduct and progress of the clinical trial. They will meet on a regular basis to ensure that there is a forum for identifying and addressing issues; and more frequently on an informal basis if required. See minutes template (Attachment 13). The TMG will pay particular attention to progress towards clinical trial milestones (recruitment, timelines etc.); adherence to the protocol; and adherence to good research practices. The TMG is responsible for reviewing the safety information to identify any serious emerging safety concerns. If safety concerns are identified, this body, in consultation with Dr Ashish Diwan (Sponsor's Independent Physician/Medical Expert) will establish a plan to minimise the time participants may be placed at excess risk of harm. Before implementing the plan, the TMG must seek the advice of the human research ethics committee and sponsor's delegate.

b) Annual assessment of safety

The following information must be provided in a report to the sponsors delegate annually:

- Documented evidence that the Trial Management Group, Trial Safety Committee, or the Data Safety Monitoring Board (e.g. meeting minutes) confirmed that regular safety reviews occurred.
- Analysis of the trial intervention(s) and its implications for participants considering all available safety data and relevant clinical or non-clinical studies results.
- Any reports of emerging safety issues and a description of any measures taken or proposed to minimise risks.
- A copy of the safety monitoring register.

10. Non-compliance, Protocol Deviation and Serious Breaches of Good Clinical Practice

10.1 Protocol Deviation

A protocol deviation is defined as any breach, divergence or departure from the requirements of Good Clinical Practice, the clinical trial protocol, the clinical trial standard operating procedures, or the human ethics approval that does not have a significant impact on the continued safety or rights of participants or the reliability and robustness of the data generated in the research or clinical trial. Protocol deviations are events that do not occur persistently or systematically and do not potentially result in participant harms. Examples of protocol deviations include but are not limited to:

- Deviations because of participant adherence to the protocol, including rescheduled study visits, participants refusal to complete scheduled research activities or failure to complete self-report questionnaires required by the study protocol.
- Blood samples obtained or clinical trial testing occurring at times close to, but not precisely at the time points specified in the protocol.
- The completion of consent forms, safety monitoring report, case report forms or data collection tools in a manner that is not consistent with the protocol instructions or failure to make reports within the required reporting timeframes.
- Administration of the clinical trial investigational medical product or device in a manner that is not consistent with the manufacturer's instructions for use.
- Use of an unapproved version of the participant information statement or recruitment of participants using unapproved recruitment procedures.
- Inclusion of a participant that does not meet the inclusion criteria.
- An urgent safety measure must be taken to eliminate an immediate hazard to a participant's health or safety.

10.2 Serious Breach of Good Clinical Practice

A serious breach is defined as a breach of Good Clinical Practice, the clinical trial protocol, the clinical trial standard operating procedures, or the human ethics approval that is likely to affect to a significant degree the safety or rights of participants or the reliability and robustness of the data generated in the clinical trial. Examples of serious breaches include but are not limited to:

- Persistent or systematic non-compliance with the instructions for completing consent forms, safety monitoring forms, case report forms or data collection tools that result in continued missed or incomplete data collection.
- Failure to record or report adverse events, serious adverse events, suspected unexpected serious adverse reactions, significant safety issues where urgent safety measures were implemented.
- Failure to conduct clinical trial procedures following the clinical trial delegation log.
- Widespread and uncontrolled use of protocol waivers affecting eligibility criteria, which leads to harm to trial subjects.
- Failure to report investigational medical product or device defects to the clinical trial sponsor or any relevant regulatory body.
- Failure to conduct research following the issued approvals, permits or licences by required laws, regulations, disciplinary standards, and UNSW policies relating to the responsible or safe conduct of research.
- Concealing or facilitating breaches (or potential breaches) of the Research Code by others.
- Researching without the requisite approvals, permits or licences required by laws, regulations, disciplinary standards, and UNSW policies related to the responsible or safe conduct of research.
- Failure to conduct research as approved by an ethics review body where that conduct leads to (or has the potential to) results in participant harms.
- Researching without ethics approval as required by the National Statement on Ethical Conduct in Human Research where that conduct leads to (or has the potential to) result in participant harms.
- Any breaches as outlined in the UNSW Research Misconduct Procedure or the Australian Code for responsible conduct of research that leads to (or can potentially) result in participant harms.

10.3 Reporting Protocol Deviations

- Protocol deviations occurring at a site must be documented in site files and reported by the principal site investigator to the Coordinating Principal Investigator.
- The Coordinating Principal Investigator must review the protocol deviation and the clinical trial protocol to establish the corrective actions and preventative steps to prevent the deviation from reoccurring.
- The protocol deviation and corrective action plan must be reported to the UNSW Sponsor's Delegate by the Coordinating Principal Investigator or Coordinating Research Team using the protocol deviation report form.

10.4 Reporting of a Serious Breach

- The Principal Investigator must report a serious breach occurring at a participating site to the Coordinating Principal Investigator within a specified timeframe.
- The Coordinating Principal Investigator must review the serious breach, along with the clinical trial protocol, to develop a Corrective and Preventive Action (CAPA) that defines the steps to prevent the serious breach from reoccurring.
- The serious breach report and the CAPA must be provided to the approving HREC, and the UNSW sponsors delegate for review and approval.

10.5 Reporting of Serious Breaches by Third Parties

- A Suspected Breach is a report judged by the reporter as a possible serious breach but has yet to be formally confirmed as a serious breach by the sponsor.
- A Suspected Breach form must be completed when a third party (e.g., individual/institution) wishes to report a suspected breach of Good Clinical Practice or the protocol and should be reported directly to the reviewing HREC without reporting through the sponsor.
- Recording of Protocol Deviation and Serious Breach Reports
- A register of protocol deviation and serious breach reports must be recorded. Written records and copies of documentation sent to the sponsor must be retained in the Investigator Site File.
- Copies of protocol deviation and serious breach reports must be recorded, written records and copies of documentation sent to the sponsor, referrals made to the HREC or establishing whether a breach of the Australian Code for Responsible conduct of research must be retained in the Master Site File.

11. Review of a Protocol Deviation and a Serious Breach

- The UNSW Sponsor's Delegate will review reports to establish whether the event meets the definition of a protocol deviation or serious breach, establish whether the proposed CAPA is appropriate and establish whether there is or will be ongoing impact reliability and robustness of the data generated.
- The UNSW Sponsor's Delegate will seek advice from the approving HREC on the corrective and preventive actions.
- Protocol deviation or serious breach reports where a UNSW researcher, staff or student is responsible for the protocol deviation or the serious breach will be reviewed as per the UNSW Research Misconduct Procedure to establish a breach of the UNSW Research Code of Conduct has occurred.
- Protocol deviation or serious breach reports where the UNSW Sponsor's Delegate determines that site personnel are responsible for a protocol deviation or the serious breach will be referred onto their responsible institution for review under their Research Misconduct procedures to establish whether a breach of the Australian Research Code for the Responsible Conduct of Research has occurred.

12. Statistics

- Describe the statistical plan for analysing the trial data.

Data Analysis Plan: For all primary and secondary outcomes that are normally-distributed and measured over time, a linear mixed model with random effect of individuals will be specified. Fixed effects of time, treatment group and the interaction will be included. Treatment differences at 9 weeks and at 21 weeks and change from baseline to 9 and 21 weeks within each group will be estimated. If the distributional assumption of normality is not tenable, an appropriate transformation of the outcome will be conducted. Alternatively, a generalized linear mixed model with appropriate distribution and link will be used.

The data will be analysed on an intention-to-treat basis. Unadjusted and adjusted analyses will be conducted. Adjusted analyses will include age at baseline and gender. P-values <0.05 will be considered significant.

MRI Data Analysis Plan: Quantification of neurotransmitter ratios of the acquired spectra will be analysed using the Java-based magnetic resonance user's interface (jMRUI 6.0, European Union project). To examine differences between periods, 2 Time (Baseline, Post) repeated-measures (RM)-ANOVA on neurotransmitter ratios will be used ($p<0.05$). Blood oxygen level

dependent (BOLD) imaging, Structural Imaging and Quantitative Arterial Spin Labelling (QASL) will be analysed using Statistical Parametric Mapping (SPM12). Significant differences between periods, 2 Time (Baseline, Post) will be determined using a random effects analysis, false discovery rate, $p < 0.05$.

We also aim to determine necessary amendments to the iDBT-Pain skills training based on the qualitative feedback from the interview with the treatment group. This data will be analysed by the investigators and summarised on a form for future development and iterations of the iDBT-Pain intervention.

- Indicate the timing of any planned interim analyses.

There is no planned interim analyses.

13. Data Ownership

All research data collected during this trial is governed and handled following the Research Data Governance and Materials Handling policy. UNSW, rather than any individual or Organisational Unit, is the Custodian of data and materials and any information derived from the data. Original research data and primary materials generated in the research conducted at the University will be owned and retained by the University subject to any contractual, statutory, ethical, or funding body requirements.

14. Handling and Reporting Data

Principal Investigators are responsible for maintaining adequate and accurate source documents and trial records that include all pertinent observations on each site's trial subjects. Source data must be attributable, legible, contemporaneous, original, accurate, and complete.

Potential participants will be assigned a unique number when they undertake the study screening (see Section 7.4). This participant ID will be automatically assigned by Qualtrics when the applicant starts the online screening.

This participant ID will be linked to the participant's direct (e.g., name, address, email address, phone #) and indirect (e.g., date of birth, sex) identifiers in a single excel password-protected database. The database will be stored separately to the deidentified data on the UNSW-supported secure OneDrive data platform. Authenticated access to UNSW OneDrive/teams folder is via zID and password.

Identifiable information about participants (e.g., name, email) and participant data (demographics; questionnaires and surveys) will be self-reported and recorded online directly to Qualtrics. Qualtrics is a data capture software that ensures curation of data via the use of standardised data collection. Its electronic capture and pre-defined fields ensure data consistency and integrity. Qualtrics uses Transport Layer Security (TLS) encryption (also known as HTTPS) for all transmitted data. The data is stored on a secure server that is hosted in a trusted data centre in Sydney (the data centres are independently audited using the industry standard SSAE-18 method). The server is protected by high-end firewall systems. Backups are performed daily. Qualtrics is recommend by UNSW for sensitive data as captured in this trial, and UNSW holds an institutional license for Qualtrics. Qualtrics is password protected via zID and password.

Only the research staff who interact with participants, and the Sponsors Independent Medical Expert (when necessary), will have access to personally identifiable information in Qualtrics and to identifiable information on the OneDrive/teams folders. All other investigators will identify participants via unique participant IDs only. All data, including brain imaging, will be coded with the unique study identifier code so that any data collected will only be identifiable by the code. All analyses will be done using de-identified data. Results will be disseminated using group data to ensure confidentiality is preserved.

Data added by members of the research team to Qualtrics derived from source documents, should be consistent with the source documents, or the discrepancies must be explained. Any change or correction to a Qualtrics entry should be dated, initialled, and explained (if necessary) and should not obscure the original entry (i.e., an audit trail should be maintained); this applies to both written and electronic changes or corrections.

The iDBT-Pain sessions will be administered via Zoom. The iDBT-Pain sessions conducted via Zoom will be recorded with the recorded files saved to a password protected server at UNSW. Recorded sessions will be only available for the researchers, and, in the case that a session is missed, will be sent to participants by email, from this trial only. This will be described to participants in the PISCF. To ensure participant cybersecurity and prevent potential hacking into the Zoom sessions, access to the Zoom sessions will be password protected. Moreover, the waiting room function within Zoom will be utilised meaning that the skills trainer will need to give each participant access to the session further securing against hacking.

De-identified imaging will be stored on a password protected UNSW secure server, with access restricted to the iDBT-Pain research team as nominated by the coordinating principal investigator. These data units are not associated with any participant identifying information. Hard copy data will be stored securely within locked filing cabinets in a locked office in the Biolink Building, School of Psychology, UNSW. It will be kept in separate files/cabinets to those containing participant details and trial identification numbers. Only approved iDBT-Pain researchers will have access to this information. If problems with data consistency are identified, audits will be conducted at the advice of the Trial Management Group.

14.1 Direct Access to Source Data and Documents

Site principal investigator(s) and institution(s) will permit trial-related monitoring, audits, IRB/IEC review, and regulatory inspection(s), providing direct access to source data/documents.

15. Monitoring Quality Control and Quality Assurance

The Coordinating Principal Investigator and Principal Investigator(s) 'responsibility are to monitor the clinical trial. The Coordinating Principal Investigator and Principal Investigator(s) are responsible for undertaking or participating in site initiation or protocol-specific training before recruitment and data collection commences. A monitoring report demonstrating regular compliance monitoring with the clinical trial protocol, procedures, and HREC approval is provided to the UNSW Sponsor's Delegate annually.
Root, cause, analysis reports are to be completed by the Coordinating Principal Investigator for reports of non-compliance and serious breaches. A corrective and preventative action plan must be developed and actioned for any reports of non-compliance and serious breaches.

16. Clinical Trial Research Agreement

The Coordinating Principal investigators must ensure that agreements are executed at each of the following sites before site initiation, recruitment, and data collection commences.

17. Research Governance Site Authorisation

Site authorisation is to be obtained, or if a research site is added, a site authorisation letter from the delegated authority of an institution responsible for any participating site is obtained. It is to be stored as a GCP essential document before participants are recruited at a participating site.

18. Good Clinical Practice Requirements

It is recommended that the Coordinating and Principal Investigators ensure that all investigators and trial-related staff have current Good Clinical Practice Training. Once completed, the evidence of training confirmation is to be stored as a GCP essential document. ICH GCP certificates for the research team will be stored as an essential document in the electronic Trial Master File (eTMF).

It is the responsibility of the Coordinating and Principal Investigators to familiarise themselves with the requirements of the Guideline for Good Clinical Practice (E6, R2)

19. Essential Documents for the Conduct of a Clinical Trial

All essential documents referred to in section 8.2 of the Guideline for Good Clinical Practice (E6, R2) are to be retained by all trial investigators.

Qualifications and Curriculum Vitae

The co-ordinating principal investigator's CV will be stored as an essential document in the electronic Trial Master File(eTMF). The eTMF will be stored on the UNSW-supported secure OneDrive data platform. Authenticated access to the UNSW OneDrive/teams folder is via zID and password. Access to the UNSW OneDrive/teams folder is restricted to the IDBT-Pain research team as nominated by the coordinating principal investigator.

20. Clinical Trial Delegation and Responsibilities Log

**Protocol / Study Number:**		**Sponsor Name:**	UNSW
**Principal Investigator Name:**	Associate Professor Sylvia Gustin	**Site Number:**	
**Site Name (if applicable)**			

***THIS FORM IS TO BE COMPLETED BY ALL PERSONNEL INVOLVED IN THE STUDY AFTER RECEIVING PROPER STUDY TRAINING AND BEFORE TAKING PART IN ANY STUDY ACTIVITIES**

Principal Investigator (PI)

By signing, I confirm/acknowledge that the tasks listed below will only be delegated to appropriately trained, skilled and qualified staff. I will remain responsible for the overall study conduct and reported data, ensuring study oversight. All associates, colleagues, and employees assisting in the conduct of the study are informed about their obligations and have not performed any study tasks before appropriate delegation and completion of appropriate training. Mechanisms are in place to ensure that site staff receives the appropriate information and training throughout the study and that a 2-way communication channel exists between staff and self. Any changes in staff or delegation in staff will be recorded promptly.

Name	Principal Investigator's Signature	Initials	Start (dd/mmm/yyyy)	End (dd/mmm/yyyy) (complete only if prior to end of study)

Site Staff

Name	Signature	Initials	Study Role	Key Study Task(s) (choose from list below)	Start (dd/mmm/yyyy)	End (dd/mmm/yyyy) (complete only if prior to end of study)	PI Initials & Date (dd/mmm/yyyy)
							________________ __/__/____
							________________ __/__/____
							________________ __/__/____
							________________ __/__/____
							________________ __/__/____
							________________ __/__/____
							________________ __/__/____
							________________ __/__/____
							________________ __/__/____

Name	Signature	Initials	Study Role	Key Study Task(s) (choose from list below)	Start (dd/mmm/yyyy)	End (dd/mmm/yyyy) (complete only if prior to end of study)	PI Initials & Date (dd/mmm/yyyy)
							______________ __/___/_____
							______________ __/___/_____
							______________ __/___/_____

Comments:

Electronic Signature Declaration for Principal Investigator and Site Staff

- My electronic signature as it applies to entering electronic data or signing records in sponsor-owned or sponsor -outsourced computer systems is the legally binding equivalent of my handwritten signature.
- I will not share password(s) assigned to me for this study with any other persons.

Principal Investigator's End of Study Declaration

I hereby confirm that the above information is accurate and complete, and that I authorised the delegation of study-related tasks to each individual as listed above.

Principal Investigator's Signature: ____ **Date:**

Task Key:

1. Obtain informed consent *
2. Subject selection/recruitment*
3. Confirm eligibility (review inclusion/exclusion criteria)*
4. Obtain medical history (source documents)
5. Perform physical exam*
6. Conduct study visit procedure as outlined in the protocol*
7. Make study-related medical decisions*
8. Assess AEs/SAEs*
9. Dispense study drug*
10. Perform drug accountability
11. Study drug storage and temperature monitoring
12. Sample collection
13. Sample processing and/or shipment
14. Evaluate study-related test results *
15. Use IWRS/IVRS
16. Make entries/corrections on (e)CRFs
17. Sign- off (e)CRFs*
18. Maintain essential documents
19. Perform study-related assessments as per protocol *
20. Complete company- specific log (if applicable)
21. Other (specify)____
22. Other (specify) ____

*These tasks may only be performed by qualified individual as permitted by local law, medical or standard of care practices, or applicable required training as per job description or designation.

21. Safety Monitoring Register

Please see Attachment 11 for the Safety monitoring form and Attachment 12 for the Adverse Events form.
