# Supplementary material for: Online Dialectical Behavioral Therapy for Emotion Dysregulation in People With Chronic Pain: A Randomized Clinical Trial
Source: JAMA Netw Open. 2025 May 6;8(5):e256908. doi: 10.1001/jamanetworkopen.2025.6908 (PMC12056567; doi:10.1001/jamanetworkopen.2025.6908)
Supplement: Supplement 2. — eMethods. eTable 1. Online Dialectical Behavioral Therapy Session Details and Homework Tasks eResults. eTable 2. Between Group Difference at 9- and 21-Weeks eTable 3. Within Group Change at 9- and 21-Weeks eReferences [file jamanetwopen-e256908-s002.pdf]

Supplemental Online Content

Norman-Nott N, Briggs NE, Hesam-Shariati N, et al. Online dialectical behavioral therapy for emotion dysregulation in people with chronic pain: a randomized clinical trial. *JAMA Netw Open*. Published online May 6, 2025. doi:10.1001/jamanetworkopen.2025.6908

eMethods.

eTable 1. Online Dialectical Behavioral Therapy Session Details and Homework Tasks

eResults.

eTable 2. Between Group Difference at 9- and 21-Weeks

eTable 3. Within Group Change at 9- and 21-Weeks

eReferences.

This supplemental material has been provided by the authors to give readers additional information about their work.

© 2025 Norman-Nott N et al. *JAMA Network Open*

eMethods

Further details about the iDBT-Pain Intervention: Pain related concerns were brought into the iDBT-Pain intervention throughout the sessions, app and handbook, and were a primary feature of discussions regarding the practical application of the DBT skills for people with chronic pain. Pain science education incorporated into the DBT modules, based on evidence about chronic pain development, the brain's role in emotional processing, and how neuroplasticity, through psychological interventions, can help unlearn pain signals over time[1,2]. Additionally, DBT skills were adapted to address pain related concerns, by enhancing mindfulness to help observe pain non-judgmentally. Drawing on Milton Erickson's work on interpreting pain[3], participants were invited to pick a name or color for their pain to foster objective observation whilst practicing the DBT skills in mindfulness. Emotion regulation and distress tolerance skills were also tailored with pain-specific examples, such as illustrating how anger can increase pain intensity in the emotion regulation module. This approach emphasized the role of DBT skills in managing emotions to potentially influence chronic pain and made the skills more relevant to this population.

© 2025 Norman-Nott N et al. *JAMA Network Open*

eTable 1. Overview of the guided sessions and self-directed learning

Topic (session)	Guided Sessions	Self-Directed Learning
Introduction (1)	Share overview of program content and meet the trainers and other participants. Introduce the concept of the connection between pain, emotions and the brain and reversing the cycle of negative emotions and pain.	*Handbook:* Read chapter 1, Introduction: Overview of the intervention; The Connection Between Pain, Emotions, and the Brain; Reversing the Cycle of Negative Emotions and Pain.
Mindfulness (2)	Present the rationale for mindfulness for chronic pain. Train and discuss introductory mindfulness skills: "wise mind", "what" skills (observing, describing, and participating).	*Handbook:* Read chapter 2, Beginning mindfulness: Evidence for Mindfulness; Mindfulness Explained; Goals of Mindfulness; Core Mindfulness Skills.<br>*App:* Watch beginning mindfulness videos and complete "wise mind", "what" skills exercises.
Mindfulness (3)	Recap and discuss mindfulness skills "wise mind" and "what skills" skills. Train and discuss mindfulness "how" skills: "non-judgementally", "one-mind" and "effectively". Complete loving kindness exercise and discuss.	*Handbook:* Read chapter 3, Beginning Mindfulness: Mindfulness "how" skills; Practicing Loving Kindness. Complete Loving kindness exercise.<br>*App:* Watch mindfulness videos and complete "how" skills exercise.
Emotion Regulation (4)	Revisit chronic pain, emotions and the brain. Introduce concepts of understanding emotions, naming emotions, and purpose of emotions. Complete in session task to identify, label and unravel negative emotional reactions. Discuss reactions and outcomes of this task.	*Handbook:* Read chapter 4, Beginning Emotion Regulation: Emotions, Chronic Pain, and the Brain; Understanding Emotions; Naming Emotions; Purpose of Emotions.<br>*App:* Watch naming emotion regulation video and complete "understanding and naming emotions" exercise.
Emotion Regulation (5)	Train and discuss changing emotions skills: "check the facts", "opposite action", and "problem solving". Discuss ways to apply changing emotion skills to the real-world.	*Handbook:* Read chapter 5, Progressing Emotion Regulation: Changing Emotional Responses; Emotion regulation skills, "Check the Facts", "Opposite Action", and "Problem Solving".<br>*App:* Watch progressing emotion regulation videos and complete "changing emotions" and "reducing negative emotions" exercises.
Emotion Regulation (6)	Introduce the importance of identifying values and goals in emotional regulation. Train and discuss skills in emotional vulnerability: "accumulate positive emotions", "build mastery", "cope ahead", and "taking care".	*Handbook:* Read chapter 6, Enhancing Emotion Regulation: Reducing Vulnerability to Painful Emotions; Accumulating Positive Emotions; Emotion regulation skills, "build mastery", "cope ahead", and "taking care".<br>*App:* Watch enhancing emotion regulation videos and complete "changing emotions", and "reducing negative emotions" exercises.
Distress Tolerance (7)	Train and discuss how to use skills to manage emotions in a crisis: "changing body chemistry", "distraction", "self-sooth" and, "pros and cons".	*Handbook:* Read chapter 7, Distress Tolerance Skills, "TIP Skills – Changing Your Body's Chemistry", "Distracting", "Pros and cons", and "Self-Soothe".<br>*App:* Watch distress tolerance videos and complete "cope ahead", "TIPs", and "distract" exercises.

© 2025 Norman-Nott N et al. *JAMA Network Open*

Topic (session)	Guided Sessions	Self-Directed Learning
Conclusion (8)	Present an overview of all skills learnt and have an open discussion about how to use them in different situations and how to maintain practice.	*Handbook:* Go back through content to revise and refresh learnings from all chapters.<br>*App:* Rewatch videos and complete exercises from each section in "mindfulness", "emotion regulation" and "distress tolerance".

Demographic data collection. At baseline, participants self-reported pain medication, and psychological therapy being received, alongside demographic and clinical data including ethnicity, education, age, sex, employment, marital status, and chronic pain condition (see Table 1 in manuscript). Ethnicity was classified by the participants and was reported to gather understand the representativeness of the sample in relation to the general population.

Further details about the primary outcome measure. The DERS-18 is a comprehensive self-report short-form of the DERS-36 designed to assess difficulties in regulating emotions[4]; validated in a chronic pain population[5]. The DERS-18 exhibited good internal consistency, reliability and convergent validity with scores highly correlated with the DERS-36, demonstrating very good concurrent validity[6].

Details about the secondary outcome measures. Secondary outcomes were pain intensity measured using the Numeric Rating Scale (range, [no pain] 0-10 [worst pain imaginable]) over the last seven days[7], pain interference measured using the 8-item Patient-Reported Outcomes Measurement Information System Pain Interference instrument (standardized score range, 40.7-77; higher scores indicate greater pain interference)[8], depression symptoms measured using the 21-item Beck Depression Inventory-2[9] (range, 0-63, higher scores indicate greater depression symptoms), anxiety symptoms measured using the 20-item State Anxiety Inventory (range, 20-80; higher scores indicate greater anxiety symptoms)[10], stress measured using the 10-item National Institutes of Health Toolbox Perceived Stress Scale (standardized score range, 22.7-87.1; higher scores indicate greater perceived stress[11], sleep problems measured using the 9-item Medical Outcomes Study Sleep Scale (range, 0-100; higher scores indicate greater sleep problems)[12], wellbeing measured using the 26-item Composure, Own-Worth, Mastery, Positivity, Achievement, Satisfaction With Life–Well-being Scale (range, 26-130; higher scores indicate better wellbeing)[13], and post-traumatic stress symptoms (PTSS) measured using the 17-item Post Traumatic Stress Disorder Checklist-Civilian Version (range, 17-85; higher scores indicate greater PTSS symptoms)[14].

© 2025 Norman-Nott N et al. *JAMA Network Open*

eResults

Sensitivity analysis: Multiple Imputation assuming Missing at Random data. As a sensitivity analysis, multiple imputation (MI) by chained equations was performed to estimate the primary outcome assuming data were missing at random (MAR). The imputation model included group, time, group by time interaction, age and sex. Other outcome variables were also imputed. They were: pain intensity, pain interference, depression, anxiety, wellbeing, post-traumatic stress symptoms, stress and sleep problems. To account for correlation due to repeated measures, imputation was performed using the "2l.pan" method within the mice package[15] in R[16]. Twenty-five data sets were imputed, and the primary outcome estimate, effect size, confidence intervals and p-values were calculated using Rubin's rules[17]. There were 2 of 44 (5%) missing values at 9-weeks in the TAU group. There were 8 of 45 (18%) missing values at 9-weeks in the intervention group. Imputation results showed a p-value for the group*time interaction of = 0.064 The group difference in change from baseline to 9-weeks was -4.403 with 95% CI -9.1 to 0.294, p = 0.066

Sensitivity analysis: Tipping point analysis assuming Missing Not at Random data. It is possible that the missing data is missing not at random (MNAR). It is plausible that those in the iDBT-Pain group who did not return for the 9-week timepoint showed a diminished change relative to what the MI analysis suggested. Therefore, we also ran a tipping point analysis to examine the effect on the primary outcome (group difference in change) with the assumption that those in the intervention group showed shallower change from baseline to 9-weeks. Using the imputed datasets, individual values for the difference from baseline to post were calculated for those in the intervention group who had an imputed value for the post time point. Then, this difference was diminished by systematically increasing values of δ of .1 to 1.0 and updated post values calculated. The estimated group difference in change and the interaction were estimated by the original model, and aggregated by Rubin's rules as in the MI analysis. Results showed the estimates for the group difference in change ranged from -4.349 (95% CI: -8.97 to 0.272; p = 0.065) to -3.918 (95% CI: -9.1 to 0.409; p = 0.075) when there was no change in the cases with imputed data.

© 2025 Norman-Nott N et al. *JAMA Network Open*

eTable 2. Between-group difference at 9- and 21-weeks

	Mean (SD)		Between-group difference		
**Outcome, timepoint**	**Control**	**Intervention**	**Estimate (95% CI)[a]**	**Cohen *d* (95% CI)[b]**	***P* value**
**Emotion Dysregulation (DERS-18)**					
Baseline	43.2 (11.1)	41.0 (10.2)	NA	NA	NA
9-week assessment	40.9 (11.2)	34.2 (7.3)	-7.1 (-11.4, -2.8)	-0.7 (-1.1, -0.3)	.001
21-week assessment	40.6 (10.9)	33.3 (7.6)	-7.6 (-12.0, -3.2)	-0.7 (-1.2, -0.3)	<.001
**Depression (BDI)**					
Baseline	19.3 (10.6)	19.0 (10.4)	NA	NA	NA
9-week assessment	23.3 (13.4)	14 (11.7)	-9.2(-14.9, -3.5)	-0.9 (-1.2, -0.3)	.002
21-week assessment	23.5 (18.0)	16.2 (12.4)	-7.5 (-13.3, -1.7)	-0.7 (-1.0, -0.1)	.01
**Anxiety (SAI)**					
Baseline	47.7 (14.2)	49.8 (11.7)	NA	NA	NA
9-week assessment	48.2 (13.3)	41.6 (13.7)	-6.5 (-11.7, -1.3)	-0.5 (-1.0, -0.1)	.02
21-week assessment	46.2 (5.4)	45.6 (4.2)	-0.9 (-6.2, 4.5)	-0.1 (-0.5, 0.4)	.74
**Stress (NIHTB-PSS)**					
Baseline	58.3 (8.7)	59.4 (8.7)	NA	NA	NA
9-week assessment	57.2 (9.7)	53.0 (7.5)	-4.6 (-8.4, -0.8)	-0.5 (-0.9, -0.1)	.02
21-week assessment	56.7 (8.8)	54.5 (7.4)	-2.4 (-6.3, 1.5)	-0.3 (-0.7, 0.2)	.22
**Post traumatic stress symptoms (PCL-C)**					
Baseline	44.3 (13.0)	44.2 (12.0)	NA	NA	NA
9-week assessment	43.8 (14.3)	36.4 (12.3)	-7.2 (-13.1, -1.3)	-0.6 (-0.9, -0.1)	.02
21-week assessment	40.2 (14.9)	35.9 (14.8)	-3.5 (-9.4, 2.5)	-0.3 (-0.7, 0.2)	.25
**Wellbeing (COMPAS-W)**					
Baseline	83.5 (12.6)	83.7 (10.7)	NA	NA	NA
9-week assessment	83.0 (13.2)	89.3 (10.3)	6.1 (0.5, 11.6)	0.5 (0.1, 1.0)	.03
21-week assessment	85.3 (14.6)	88.1 (11.4)	2.8 (-2.9, 8.4)	0.2 (-0.2, 0.7)	.34
**Sleep (MOSS)**					
Baseline	43.6 (14.7)	42.4 (16.9)	NA	NA	NA
9-week assessment	45.2 (12.8)	37.4 (14.2)	-7.9 (-14.6, -1.2)	-0.5 (-1.0, -0.1)	.02
21-week assessment	42.3 (14.8)	37.8 (11.4)	-4.7 (-11.5, 2.2)	-0.3 (-0.8, 0.1)	.18
**Pain Intensity (NRS)**					
Baseline	6.34 (1.4)	6.53 (1.4)	NA	NA	NA
9-week assessment	6.43 (1.7)	5.86 (2.1)	-0.5 (-1.3, 0.2)	-0.4 (-0.7, 0.1)	.18
21-week assessment	6.37 (2.0)	5.30 (2.0)	-1.1 (-1.9, -0.3)	-0.8 (-1.1, -0.1)	.005
**Pain Interference (PROMIS)**					
Baseline	64.5 (5.7)	64.6 (5.7)	NA	NA	NA
9-week assessment	64.8 (6.0)	63.3 (7.8)	-1.4 (-4.4, 1.6)	-0.2 (-0.7, 0.2)	.35
21-week assessment	63.9 (7.1)	62.7 (6.4)	-1.2 (-4.2, 1.8)	-0.2 (-0.7, 0.3)	.43

Abbreviations: BDI = Beck Depression Index (range, 0-63, higher scores indicate greater depression symptoms); COMPAS-W = Composure, Own-Worth, Mastery, Positivity, Achievement, Satisfaction With Life–Well-being Scale (range, 26-130; higher scores indicate better wellbeing); DERS-18 = Difficulties in Emotion Regulation Scale (range, 18-90; higher scores indicate higher emotion dysregulation); MOS-SS = Medical Outcomes Study Sleep Scale (range, 0-100; higher scores indicate greater sleep problems); NA =, not applicable; NIHTB-PSS = National Institutes of Health Toolbox Perceived Stress Scale (standardized score range, 22.7-87.1; higher scores indicate greater perceived stress); NRS = Numeric Rating Scale (range, [no pain] 0-10 [worst pain imaginable]); PROMIS = Patient-Reported Outcomes Measurement Information System Pain Interference instrument (standardized score range, 40.7-77; higher scores indicate greater pain interference); PCL-C = Post Traumatic Stress Disorder Checklist-Civilian Version (range, 17-85; higher scores indicate greater symptoms); SAI = State Anxiety Inventory (range, 20-80; higher scores indicate greater anxiety symptoms).

[a]Between group difference from the linear mixed model, including all available data.

[b]Effect size estimates calculated from scaling the pooled baseline standard deviation of the outcome with confidence intervals estimated from bootstrapped percentile values from 1000 resamples.

© 2025 Norman-Nott N et al. *JAMA Network Open*

eTable 3. Within-group change at 9- and 21-weeks

	Mean (SD)			Within-group change (baseline to 9-weeks)			Within-group change (baseline to 21-weeks)		
**Outcome, timepoint**	**Baseline**	**9-weeks**	**21-weeks**	**Estimate (95% CI)[a]**	**Cohen *d* (95% CI)[b]**	***P* value**	**Estimate (95% CI)[a]**	**Cohen *d* (95% CI)[b]**	***P* value**
**Emotion Dysregulation (DERS-18)**									
Control	43.2 (11.1)	40.9 (11.2)	40.6 (10.9)	-2.2 (-5.2, 0.8)	-0.2 (-0.5, 0.1)	.14	-2.6 (-5.7, 0.5)	-0.2 (-0.5, 0.0)	.10
Intervention	41.0 (10.2)	34.2 (7.3)	33.3 (7.59)	-7.1 (-10.2, -4.0)	-0.7 (-1.0, -0.4)	< .001	-8.0 (-11.1, -4.9)	-0.8 (-1.1, -0.5)	<.001
**Depression (BDI)**									
Control	19.3 (10.6)	23.3 (13.4)	23.5 (18.0)	4.0 (0.3, 7.5)	0.4 (0.0, 0.6)	.03	3.8 (0.1, 7.5)	0.4 (0.0, 0.6)	.04
Intervention	19.0 (10.4)	14 (11.7)	16.2 (12.4)	-4.9 (-8.5, -1.3)	-0.47 (-0.7, -0.1)	.007	-3.3 (-7.0, 0.3)	-0.3 (-0.6, 0.0)	.07
**Anxiety (SAI)**									
Control	47.7 (14.2)	48.2 (13.3)	46.2 (5.36)	0.6 (-3.2, 4.4)	0.05 (-0.3, 0.4)	.76	-1.9 (-5.9, 2.0)	-0.2 (-0.5, 0.2)	.33
Intervention	49.8 (11.7)	41.6 (13.7)	45.6 (4.17)	-7.8 (-11.6, -4.0)	-0.6 (-1.0, -0.4)	< .001	-4.8 (-8.7, -0.9)	-0.4 (-0.7, -0.1)	.02
**Stress (NIHTB-PSS)**									
Control	58.3 (8.7)	57.2 (9.7)	56.7 (8.8)	-0.5 (-3.1, 2.1)	-0.06 (-0.4, 0.2)	.70	-1.5 (-4.2, 1.1)	-0.2 (-0.5, 0.1)	.25
Intervention	59.4 (8.7)	53.0 (7.5)	54.5 (7.4)	-6.1 (-8.7, -3.6)	-0.7 (-1.0, -0.4)	<.001	-5.0 (-7.6, -2.3)	-0.6 (-0.8, -0.3)	<.001
**Post traumatic stress symptoms (PCL-C)**									
Control	44.3 (13.0)	43.8 (14.3)	40.2 (14.9)	-1.1 (-4.5, 2.3)	-0.1(-0.3, 0.2)	.54	-4.8 (-8.3, -1.4)	-0.4 (-0.6, -0.1)	.006
Intervention	44.2 (12.0)	36.4 (12.3)	35.9 (14.8)	-8.0 (-11.4, -4.6)	-0.6 (-0.9, -0.3)	<.001	-8.0 (-11.4, -4.6)	-0.6 (-0.8, -0.3)	<.001
**Wellbeing (COMPAS-W)**									
Control	83.5 (12.6)	83.0 (13.2)	85.3 (14.6)	-1.2 (-4.6, 2.3)	-0.1 (-0.4, 0.2)	.50	2.1 (-1.4, 5.5)	0.2(-0.1, 0.5)	.25
Intervention	83.7 (10.7)	89.3 (10.3)	88.1 (11.4)	4.6 (1.2, 8.0)	0.4 (0.1, 0.7)	.008	4.5 (1.0, 8.0)	0.4 (0.1, 0.6)	.01
**Sleep (MOSS)**									
Control	43.6 (14.7)	45.2 (12.8)	42.3 (14.8)	2.1 (-2.4, 6.7)	0.1 (-0.1, 0.4)	.36	-0.2 (-4.8, 4.4)	-0.0 (-0.3, 0.3)	.93
Intervention	42.4 (16.9)	37.4 (14.2)	37.8 (11.4)	-4.7(-9.2, -0.2)	-0.3 (-0.6, -0.0)	.04	-3.7 (-8.4, 1.0)	-0.2 (-0.6, 0.1)	.11
**Pain Intensity (NRS)**									
Control	6.3 (1.4)	6.4 (1.7)	6.4 (2.0)	0.1 (-0.5, 0.7)	0.1 (-0.3, 0.4)	.72	0.1 (-0.4, 0.7)	0.1(-0.2, 0.4)	.67
Intervention	6.5 (1.4)	5.9 (2.1)	5.3 (2.0)	-0.6 (-1.2, -0.0)	-0.4 (-0.6, -0.0)	.04	-1.2 (-1.8, -0.6)	-0.9 (-2.0, -0.3)	<.001
**Pain Interference (PROMIS)**									
Control	64.5 (5.7)	64.8 (6.0)	63.9 (7.1)	0.3 (-1.9, 2.4)	0.05 (-0.3, 0.4)	.80	-0.5 (-2.7, 1.7)	-0.1 (-0.4, 0.3)	.66
Intervention	64.6 (5.7)	63.3 (7.8)	62.7 (6.4)	-1.2 (-3.3, 0.9)	-0.2 (-0.5, 0.1)	.27	-1.8 (-3.9, 0.4)	-0.3 (-0.6, 0.1)	.11

Abbreviations: BDI = Beck Depression Index (range, 0-63, higher scores indicate greater depression symptoms); COMPAS-W = Composure, Own-Worth, Mastery, Positivity, Achievement, Satisfaction With Life–Well-being Scale (range, 26-130; higher scores indicate better wellbeing); DERS-18 = Difficulties in Emotion Regulation Scale (range, 18-90; higher scores indicate higher

© 2025 Norman-Nott N et al. *JAMA Network Open*

emotion dysregulation); MOS-SS = Medical Outcomes Study Sleep Scale (range, 0-100; higher scores indicate greater sleep problems); NA =, not applicable; NIHTB-PSS = National Institutes of Health Toolbox Perceived Stress Scale (standardized score range, 22.7-87.1; higher scores indicate greater perceived stress); NRS = Numeric Rating Scale (range, [no pain] 0-10 [worst pain imaginable]); PROMIS = Patient-Reported Outcomes Measurement Information System Pain Interference instrument (standardized score range, 40.7-77; higher scores indicate greater pain interference); PCL-C= Post Traumatic Stress Disorder Checklist-Civilian Version (range, 17-85; higher scores indicate greater symptoms); SAI = State Anxiety Inventory (range, 20-80; higher scores indicate greater anxiety symptoms).

[a]Within group difference from the linear mixed model, including all available data.

[b]Effect size estimates calculated from scaling the pooled baseline standard deviation of the outcome with confidence intervals estimated from bootstrapped percentile values from 1000 resamples.

© 2025 Norman-Nott N et al. *JAMA Network Open*

eReferences

1. Butler DS, Moseley GL. *Explain pain*. Noigroup Publications (2003).
2. Kang D, Hesam-Shariati N, McAuley JH, et al. Disruption to normal excitatory and inhibitory function within the medial prefrontal cortex in people with chronic pain. https://doi.org/10.1002/ejp.1838. *European Journal of Pain*. 2021/11/01 2021;25(10):2242-2256. doi:https://doi.org/10.1002/ejp.1838
3. Erickson MH. An Introduction to the Study and Application of Hypnosis for Pain Control. Springer Berlin Heidelberg; 1967:83-90.
4. Gratz KL, Roemer L. Multidimensional assessment of emotion regulation and dysregulation: Development, factor structure, and initial validation of the difficulties in emotion regulation scale. *Journal of psychopathology and behavioral assessment*. 2004;26(1):41-54.
5. Kökönyei G, Urbán R, Reinhardt M, Józan A, Demetrovics Z. The difficulties in emotion regulation scale: factor structure in chronic pain patients. *J Clin Psychol*. Jun 2014;70(6):589-600. doi:10.1002/jclp.22036
6. Victor SE, Klonsky ED. Validation of a Brief Version of the Difficulties in Emotion Regulation Scale (DERS-18) in Five Samples. *Journal of psychopathology and behavioral assessment*. 2016;38(4):582-589. doi:10.1007/s10862-016-9547-9
7. Hawker GA, Mian S, Kendzerska T, French M. Measures of adult pain: Visual analog scale for pain (vas pain), numeric rating scale for pain (nrs pain), mcgill pain questionnaire (mpq), short-form mcgill pain questionnaire (sf-mpq), chronic pain grade scale (cpgs), short form-36 bodily pain scale (sf-36 bps), and measure of intermittent and constant osteoarthritis pain (icoap). *Arthritis care & research*. 2011;63(S11):S240-S252.
8. Askew RL, Cook KF, Revicki DA, Cella D, Amtmann D. Evidence from diverse clinical populations supported clinical validity of PROMIS pain interference and pain behavior. *J Clin Epidemiol*. May 2016;73:103-11. doi:10.1016/j.jclinepi.2015.08.035
9. Beck AT, Steer RA, Brown G. Beck depression inventory–II. *Psychological assessment*. 1996;doi:https://doi.org/10.1037/t00742-000
10. Spielberger CD, Gorsuch RL, Lushene R. *Manual for the State-Trait Anxiety Inventory*. Consulting Psychologists' Press; 1970.
11. Cohen S, Williamson G. Perceived stress in a probability sample of the United States. In: Spacapam S, Oskamp S, eds. *The Social Psychology of Health*. Sage; 1988: 31–67.
12. Hays RD, Stewart A. Sleep measures. In: Stewart AL, Ware JE, eds. *Measuring functioning and well-being: The Medical Outcomes Study approach (pp 235-259),* . Duke University Press; 1992:235-259.
13. Gatt JM, Burton KL, Schofield PR, Bryant RA, Williams LM. The heritability of mental health and wellbeing defined using COMPAS-W, a new composite measure of wellbeing. . *Psychiatry Research*. 2014;219(1):204-213. doi:10.1016/j.psychres.2014.04.033
14. Weathers F, Litz B, Herman D, Huska JA, Keane T. PTSD Checklist: Reliability, validity, and diagnostic utility. *Proceedings of the 9th Annual Meeting of the International Society for Traumatic Stress Studies (ISTSS)*. 01/01 1993;
15. van Buuren S, Groothuis-Oudshoorn K. mice: Multivariate Imputation by Chained Equations in R. *Journal of Statistical Software*. 12/12 2011;45(3):1 - 67. doi:10.18637/jss.v045.i03
16. *R: A Language and Environment for Statistical Computing*. R Foundation for Statistical Computing; 2024. https://www.R-project.org/
17. Rubin DB. *Multiple Imputation for Nonresponse in Surveys*. John Wiley & Sons Inc; 1987.

© 2025 Norman-Nott N et al. *JAMA Network Open*
