# Supplementary material for: Online Dialectical Behavioral Therapy for Emotion Dysregulation in People With Chronic Pain: A Randomized Clinical Trial
Source: JAMA Netw Open. 2025 May 6;8(5):e256908. doi: 10.1001/jamanetworkopen.2025.6908 (PMC12056567; doi:10.1001/jamanetworkopen.2025.6908)
Supplement: Supplement 3. — Data Sharing Statement [file jamanetwopen-e256908-s003.pdf]

Data Sharing Statement

Norman-Nott. Online Dialectical Behavioral Therapy for Emotion Dysregulation in People With Chronic Pain. *JAMA Netw Open*. Published May 06, 2025.
doi:10.1001/jamanetworkopen.2025.6908

Data
Additional Information: Australia and New Zealand Clinical Trial Registry: Anzctr.org.au. Identifier: ACTRN12622000113752
Data available: Yes
Data types: Deidentified participant data
How to access data: Anonymized data sets and associated material will be available upon reasonable request from the corresponding author: n.normannott@unsw.edu.au
When available: With publication

Supporting Documents
Document types: Other (please specify)
Additional Information: Trial Protocol and statistical analysis plan Supplemental Materials containing main effects and sensitivity analysis
How to access documents: https://www.researchprotocols.org/2023/1/e41890 Linked as a Supplemental File
When available: With publication

Additional Information
Who can access the data: Data will be made available to researchers whose proposed use of the data has been approved.
Types of analyses: Researchers can request the data for independent participant data meta-analyses.
Mechanisms of data availability: Data access will be given after approval for specific analyses, and data sharing agreements are signed.
